# Vast, overlooked peat, and organic soils in Brazil's Cerrado: carbon storage, dynamics, and stability

**DOI:** 10.1111/nph.71027

**Published:** 2026-03-12

**Authors:** Larissa S. Verona, Amy E. Zanne, Susan Trumbore, Paulo N. Bernardino, Guilherme M. Alencar, Thalia Andreuccetti, David Herrera‐Ramírez, João C. F. Cardoso, Demetrius Lira‐Martins, Guilherme G. Mazzochini, Natashi Pilon, Rafael S. Oliveira

**Affiliations:** ^1^ Programa de pós‐graduação em Biologia Vegetal, Departamento de Biologia Vegetal, Instituto de Biologia Universidade Estadual de Campinas Campinas São Paulo 13083‐862 Brazil; ^2^ Cary Institute of Ecosystems Studies Millbrook NY 12545 USA; ^3^ Max Planck Institute for Biogeochemistry Jena 07745 Germany; ^4^ Universidade Estadual de Campinas, Departamento de Biologia Vegetal Campinas São Paulo 13083‐862 Brazil; ^5^ Yale School of the Environment Yale University New Haven CT 06511 USA; ^6^ Yale Institute for Biospheric Studies Yale University New Haven CT 06520 USA; ^7^ Programa de Pós‐Graduação em Ecologia, Conservação e Manejo da Fauna Silvestre Universidade Federal de Minas Gerais Belo Horizonte 31270‐910 Brazil; ^8^ Earthshot Labs Sebastopol CA 95472 USA; ^9^ Diretoria de Pesquisa Científica Instituto de Pesquisas Jardim Botânico do Rio de Janeiro Rio de Janeiro 22460‐030 Brazil

**Keywords:** carbon cycle, climate change, methane, palm swamp, tropical peatlands, Veredas, Campos úmidos, wetlands

## Abstract

- Tropical peatlands are critical for climate mitigation due to their dual role as major carbon sinks and methane sources. In rainforests, high and stable rainfall supports peat accumulation in tropical climates. However, groundwater‐fed peatlands in seasonally dry tropical ecosystems remain poorly understood, despite their potential importance in global carbon dynamics.
- Here, we present an integrated carbon assessment in organic soil ecosystems (locally known as Veredas and Campos úmidos) in the Brazilian savanna. We quantified carbon in soil and biomass, dated carbon using radiocarbon, and evaluated chemical stability using infrared spectrometry. We used machine learning models to map their potential area. Additionally, we measured soil CO_2_ and CH_4_ efluxes to evaluate the influence of climatic seasonality on emissions.
- Veredas contained exceptionally high carbon stocks (*c.* 1200 Mg C ha^−1^) accumulated over *c.* 20 000 yr and spanning *c.* 16.7 Mha. However, spectroscopy indicated low carbon stability compared to other tropical peatlands, and *c.* 70% of annual CO_2_ and CH_4_ emissions occurred during the dry season.
- Our findings show that the Brazilian Cerrado harbors one of the largest carbon‐storing ecosystems in the tropical Americas, yet one that is highly vulnerable to land‐use change and intensified drought. Despite their wide distribution, peat accumulation and the extent of Veredas remain uncertain.

## Introduction

Carbon is a critical element on Earth, underpinning life and contributing to global warming via greenhouse gas emissions. Understanding its distribution and turnover is therefore critical (IPCC *et al*., 2022). Soils represent the largest terrestrial organic carbon reservoir, storing more carbon than the atmosphere and vegetation combined, and therefore play a pivotal role in the global carbon budget (Scharlemann *et al*., 2014; Köchy *et al*., 2015). Organic‐rich soils, such as those found in wetlands, are particularly important due to their exceptional carbon storage capacity (Köchy *et al*., 2015). These ecosystems develop under anoxic conditions, leading to large accumulations of carbon in soil as poorly decomposed biomass, potentially for millennia (Clymo, 1987; Clymo *et al*., 1998). Among them, peatlands represent an extreme end member of organic soils, characterized by exceptionally high carbon densities and increasingly recognized for their global importance (Clymo *et al*., 1998; FAO, 2020). Although there is disagreement about the carbon‐density thresholds used to classify peatlands (Lourenco *et al*., 2022), there is broad consensus that wetland ecosystems with carbon‐rich soils are among the most important components of the global carbon balance.

Peatlands cover only 3% of the Earth's terrestrial surface, but store almost 21% of the global soil carbon (Perish *et al*., 2008; Leifeld & Menichetti, 2018). Furthermore, wetlands, which include peatlands, are also the largest natural source of CH_4_, representing 20% of global emissions annually (Saunois *et al*., 2020). However, soil carbon‐rich ecosystems are under increasing threat from land‐use change and climate impacts (Frolking *et al*., 2011; Global Peatlands Initiative, 2022; IPCC *et al*., 2022). Approximately 25% of peatlands are already degraded, and 12% are no longer accumulating carbon (FAO, 2020). In these degraded areas, carbon emissions are 10× greater than carbon accumulation, contributing to *c*. 4% of global glasshouse gas (GHG) emissions (Global Peatlands Initiative, 2022). Given their importance and vulnerability, the identification, understanding, and protection of carbon‐rich soils, particularly peatlands, are priorities in climate change mitigation actions.

Peatlands have a global distribution but are most concentrated in high latitudes of the Northern Hemisphere, where cold climates favor long‐term carbon accumulation in soils (Global Peatlands Initiative, 2022). In the wet tropics, high and constant rainfall enables reduced decomposition rates even in the absence of thermal limitations, resulting in extensive peatland areas within rainforest regions (Global Peatlands Initiative, 2022). Tropical peatlands began forming *c*. 20 000 yr ago, nearly twice as early as the northern peatlands, which developed after the retreat of ice sheets (Yu *et al*., 2010; Sjögersten *et al*., 2014). In addition, peat in wet tropical regions typically contains higher concentrations of chemically stable compounds. This stability is driven both by the dominance of highly lignified vegetation typical of tropical wetlands and greater decomposition of labile carbon under persistently high temperatures (Hodgkins *et al*., 2018; Verbeke *et al*., 2022).

Understanding the stability of peat organic matter to decomposition is essential for climate change mitigation (Normand, 2017). The shifting global climate is altering temperature and precipitation regimes, leading to reduced water table levels in peatlands, thereby exposing organic carbon to oxidation (Frolking *et al*., 2011). This decomposition results in increased CO_2_ emissions to the atmosphere (Huang *et al*., 2021; Ribeiro *et al*., 2021), which in turn amplifies climate warming and increases the vulnerability of the remaining peatlands. In the face of such climatic uncertainties, mapping and conserving stable peatlands becomes a priority (Sjögersten *et al*., 2014; Leifeld & Menichetti, 2018; Strack *et al*., 2022). Despite comprising a smaller portion of global peatlands, tropical peatlands play an important role in global carbon stocks, with an estimate of *c*. 120 Gt of stored carbon (Leifeld & Menichetti, 2018). Yet their importance may be poorly underestimated due to limited research on their presence, extent, and functioning. Advancing the understanding, mapping, and conservation efforts of tropical peatlands is therefore critical not only to address climate change through nature‐based solutions but also to safeguard carbon stocks and secure the necessary investments to protect these ecosystems.

Recently there has been renewed interest in tropical peatlands, with most studies outside Asia occurring in the last decade (Draper *et al*., 2014; Dargie *et al*., 2019). These larger peat areas in the tropics occur mainly in rainforests, with high annual rainfall and low seasonality (Gumbricht *et al*., 2017; Xu *et al*., 2018; FAO, 2020; Ribeiro *et al*., 2021; Global Peatlands Initiative, 2022). Contrary to earlier assumptions, recent evidence suggests extensive peat and organic carbon accumulation in seasonally dry regions of South America, such as the Cerrado, the Brazilian savanna, maintained by shallow water table levels (Wantzen *et al*., 2012; Sousa *et al*., 2015; Soares *et al*., 2021; Horák‐Terra *et al*., 2022a, 2022b; Beer *et al*., 2024). The Cerrado is the second largest biome in South America, occupying 26% of Brazil. It is a biodiversity hotspot due to its high species richness and endemism, making it the world's most biodiverse savanna (Myers *et al*., 2000; Vieira *et al*., 2022), yet it is also highly threatened by land‐use change and large‐scale drainage of wetlands for agriculture and infrastructure expansion. In this region, Veredas (palms swamps) and campos úmidos (wet grasslands) accumulate peat, although the amount of peat they contain and their total extent are largely unknown. These ecosystems do play an important role in the Cerrado landscape, acting as water sources for biodiversity and human populations in seasonal environments (Ramos *et al*., 2006; Queiroz, 2015). Furthermore, peatlands in tropical savannas face strong climatic seasonality, which can affect flooding and carbon dynamics. Despite their potential as carbon sinks, few studies have assessed carbon stocks in Veredas (Wantzen *et al*., 2012; Sales *et al*., 2020; Soares *et al*., 2021; Horák‐Terra *et al*., 2022b; Santos *et al*., 2023; Souza *et al*., 2025). Only two studies investigated stocks deeper than 1 m (Horák‐Terra *et al*., 2022b; Santos *et al*., 2023), and just one measured soil carbon fluxes in these systems (Meirelles *et al*., 2006). As a result, the effects of Cerrado seasonality on peat accumulation, stability, and carbon fluxes remain largely unknown.

To address critical knowledge gaps in tropical peatland science, this study focuses on Veredas in the Brazilian Cerrado. We pursued four main objectives: (1) to quantify carbon stocks; (2) to determine the age and stability of stored carbon; (3) to predict total Vereda area; and (4) to assess how climatic seasonality affects carbon fluxes in Veredas. We investigated whether (1) carbon stocks in Veredas are comparable to those reported in other tropical peatlands; (2) stored peat contains old stable carbon, with maximum ages ranging from 10 000 to 40 000 calibrated years Before Present (years calBP), coinciding with the start of peat formation elsewhere in Brazil; (3) Veredas are spread throughout the entire Cerrado; and (4) a lower water table during the dry season increases GHG emissions by enhancing aerobic decomposition, that is CO_2_ loss, outweighing any reduction in CH_4_ (in CO_2_ equivalent). To test these, we sampled above and belowground biomass in Veredas across the central Cerrado, and analyzed carbon concentration, total soil volume, maximum peat age, and organic matter stability. We also predicted the total Vereda extension using machine learning. In parallel, we measured CO_2_ and CH_4_ fluxes in Veredas during wet, dry, and transitional seasons. Our findings provide key insights into carbon dynamics in tropical savanna peatlands and underscore the urgency of conserving Cerrado wetlands, particularly Veredas and Campos úmidos, as a nature‐based climate solution.

## Materials and Methods

### Study area

**Fig. 1:**
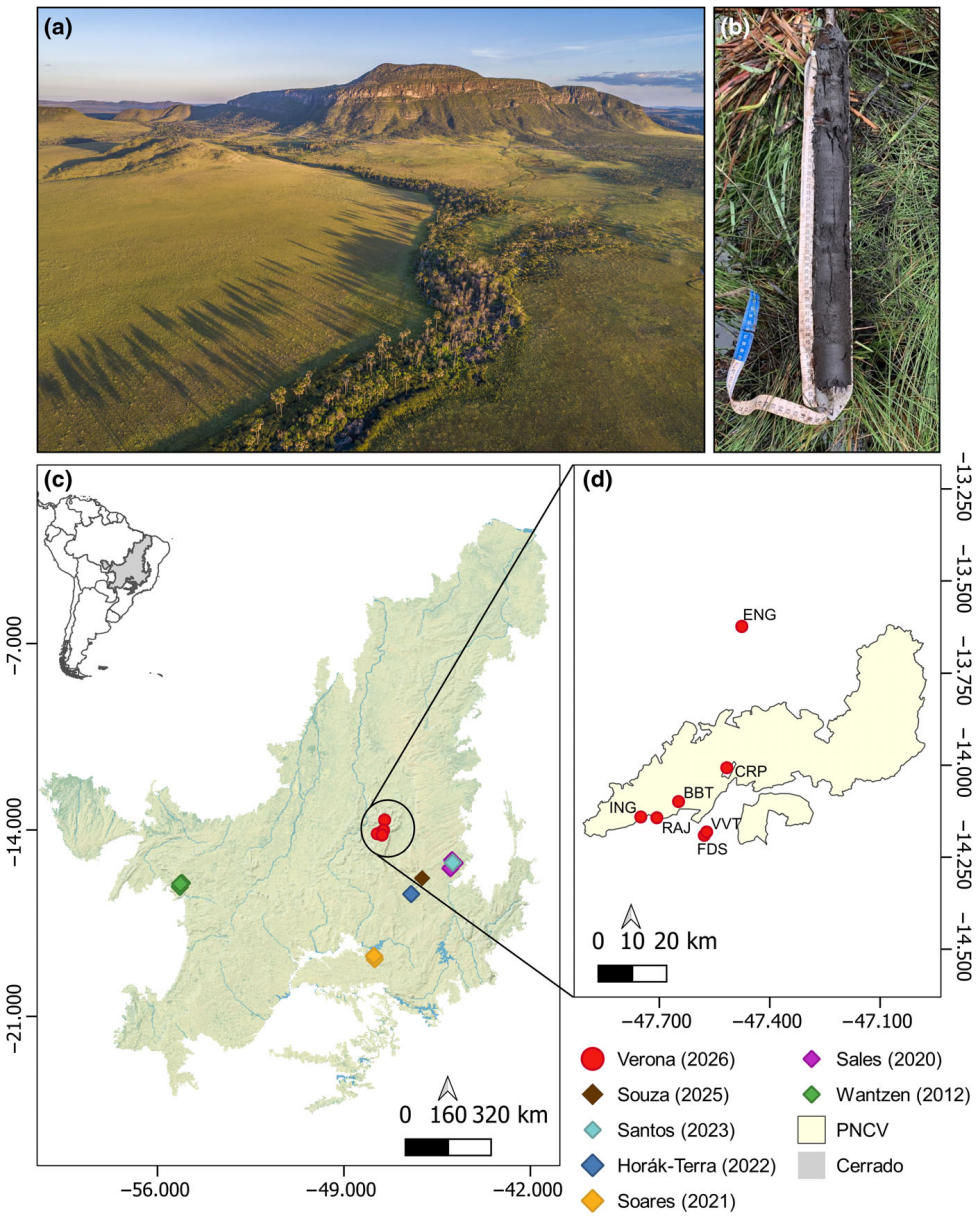
Examples of Vereda and soils, and maps showing the locations of Veredas from previous and current studies. (a) Aerial view of Vereda ecosystem in Chapada dos Veadeiros National Park, (PNCV) and (b) an example of an organic soil profile between 100 and 150 cm depth in RAJ site. (c) Location and distribution of the Cerrado in South America and studied Veredas of the current (red circles) and previous studies (colored diamonds). (d) Detail of the sampled sites of the current study. Photo credits (a) A. Dib; (b) L. Verona.

This study was conducted in seven wetland areas, including Veredas and campos úmidos, in and around Chapada dos Veadeiros National Park, Brazil (Fig. 1). Veredas are groundwater‐dependent wetlands present in Brazilian Cerrado, characterized by an herbaceous layer with a predominance of grasses and, frequently, sparse occurrence of palm *Mauritia flexuosa*, locally known as ‘Buriti’ (Ribeiro & Walter, 1998; Fig. 1a). Campos úmidos are groundwater‐dependent wet grasslands lacking palms, dominated by herbaceous vegetation and maintained by shallow water tables. Hereafter, we use the term Veredas to refer to these open, groundwater‐dependent grassland ecosystems, including both palm‐dominated *Veredas* and palm‐free campos úmidos. The dominant herbaceous species include *Axopus siccus*, *Andropogon virgatus*, *Andropogon hypogynus*, *Paspalum maculosum*, *Curtia tenuifolia*, *Tetrapollinia caerulescens*, *Microlicia psammophila*, *Drosera hirtella*, *Xyris spp*., and *Chaetogastra gracilis*. Veredas occur as linear islands on the landscape, located in shallow valleys or small depressions associated with springs and first‐order streams (Fig. 1a), coinciding with riparian areas (Ferreira, 2005). As they are groundwater‐fed wetlands, Veredas can be flooded throughout the year, and water levels are susceptible to seasonal fluctuations (Augustin *et al*., 2009). Their geological formation results from flat surfaces and layered lithified strata, with an impermeable layer below a permeable one creating the potential for perched water tables (Boaventura, 1978; Ferreira, 2005). Despite being legally protected (Law n° 12 651/2012, Native vegetation protection law; Brasil, 2012), some regions recorded up to 50% degradation of Vereda areas and their surroundings in recent decades (Brasil *et al*., 2021; Gonçalves *et al*., 2022); this value is uncertain and probably underestimated due to the lack of national information on total land conversion (Bassani *et al*., 2025).

Vereda soils can be classified as peat or organic soils (Boaventura, 1978). Although there is no consensus regarding the threshold of soil organic carbon that defines peat soils or peatlands (Lourenco *et al*., 2022), these ecosystems are generally characterized by waterlogged conditions that promote the accumulation of carbon as undecomposed organic matter. FAO, IPCC, the Global Peatland Assessment uses thresholds between 12 and 18% of soil organic C to define peatlands (IPCC, 2013; FAO, 2020; Global Peatlands Initiative, 2022). By contrast, the Brazilian Soil Classification System considers soils with > 8% organic carbon as organic (Santos *et al*., 2018). To avoid misinterpretations in the classification, we use the term ‘peat and organic soils’ to encompass the variability in soil carbon content in Veredas, thereby ensuring consistency across different contexts.

The study sites (Fig. 1c,d) contain native open wetlands with minimal disturbance, except for occasional fires typical of the Cerrado ecosystem. The sites were selected for their proximity to roads and ease of access, as well as the authorized permits to enter the areas. Two sites were located inside the park (ING and BBT sites), one was located in the Quilombo Kalunga Traditional Afro‐Brazilian descendants Territory (ENG site), and the rest were located in private properties around the park (RAJ, FDS, VVT, and CRP sites). The mean annual temperature for the areas is 21°C and the mean annual precipitation is *c*. 1400 mm, with most of the precipitation (95%) occurring during the wet season (Supporting Information Fig. S1), which lasts for 7 months (October to May; Alvares *et al*., 2013).

### Carbon stocks

#### Soil organic carbon

To calculate the total carbon stock in each wetland site, we first estimated the total soil organic carbon (SOC). To assess this, we established three transects perpendicular to the water flow at five Veredas, maintaining a minimum distance of 20 m between transects. At the VVT, the small peat area allowed for the establishment of only two transects. We ended transects at the forest border to avoid including riparian forests, which have different soil carbon characteristics.

Along each transect, we characterized belowground carbon stocks by collecting three soil cores spaced at least 6 m apart, with a mean distance of 37 m between them (two from the border and one from the center) (Fig. S2a) using a Russian Peat Auger to sample undisturbed soils for the entire carbon layer (Fig. S2d). The sum of all layer thicknesses at each point was denoted here as ‘depth’. We limited our samples to 4 m depth due to equipment limitations, although some locations had even deeper organic carbon. If a mineral layer, identified by a change in soil color, or bedrock was reached before 4 m, sampling was terminated. Around 67% of the sampling efforts were stopped before 4 m due to reaching mineral soil layers, such as gleyed soil, clay, or sand. The sampling design, consisting of transects and points, was planned to cover both the central and peripheral zones of each Vereda. Each location of a soil core is named here as a ‘point’. We visually characterized each soil profile by changes in color and texture (Fig. S2d) and collected one sample from each layer if it was smaller than 50 cm. If the layer was larger, we collected one sample for every 50 cm. Each sample had a length of 5 cm and a total volume of 49 cm^3^. A summary of soil sampling information is in Table S1.

To measure the total point carbon stock, we used the equation below:

$$C_{\text{stock}}=\Sigma \;\left(\;L_{i}*D_{i}*C_{\mathrm{org}\;i}\right)$$

The carbon stock ($C_{\text{stock}}$; kg m^−2^, later converted to Mg ha^−1^), was calculated as the sum of the carbon stock in each layer (i). For each layer, the carbon stock was obtained by multiplying the layer length ($L_{i}$; m), the bulk density ($D_{i};$ kg m^−3^), the concentration of organic carbon ($C_{\mathrm{org}\;i}$; %) (Agus *et al*., 2011; FAO, 2020). The carbon stock for each site was measured as the mean of all points. We used a null linear mixed‐effects model to estimate the total carbon stock for Veredas, treating carbon stock at each sampling point as the dependent variable and including site as a random intercept. This approach allowed us to estimate the overall mean carbon stock while accounting for variation among sites. We also estimated the maximum and minimum carbon‐density values for each site by combining the respective maximum and minimum values of carbon content, bulk density, and depth of each site.

To define the limits of each Vereda, we used remote sensing by analyzing microwave bands from Sentinel‐1 and RGB images from Google Earth Images and drawing Vereda limits manually. The microwave data allowed us to determine regions with high water content in vegetation and soil (Steele‐Dunne *et al*., 2017; Konings *et al*., 2019). We contrasted images from the dry and wet seasons to compare the elevated water limits at both extremes. Finally, we conducted a ground validation by contrasting the mapped area with the waterlogged area during the wet season.

For soil density, we dried the samples in a 60°C oven for at least 48 h and weighed them. We obtained the volume using the dimensions of the auger (half a cylinder with a 2.5 cm radius) and the length of the samples (5 cm), and calculated density based on the mass : volume ratio. We determined organic carbon using a Truspec Micro Elemental Analyzer at Luiz de Queiroz College of Agriculture, University of São Paulo. For this analysis, the samples were homogenized, sieved to 0.15 mm, and a subsample of a few milligrams was collected. Soil bulk density and organic carbon for each layer were measured from the same sample, ensuring consistency in storage estimates.

#### Biomass carbon

To determine biomass carbon stocks in Veredas, we collected the above‐ and belowground biomass for herbaceous plants and estimated biomass for non‐herbaceous plants. We established two 10 m radius plots in each transect (Fig. S2b), coincident with the central sample point and one border sample point. In each plot, we estimated the height of each non‐herbaceous plant in m with a rangefinder (SNDWAY SW‐1500A). From a fixed point at the same height as the palm base, we measured the distance to the tree base and the distance to the tree's tallest point, and calculated height using triangle similarity algorithms. The only non‐herbaceous species that occurred inside our sampled plots was *M. flexuosa* palms. The non‐herbaceous above and belowground biomass was estimated using the allometric equations below, calculated for *M. flexuosa* (Goodman *et al*., 2013).

$$\log_{e}\mathrm{AGB}=2.4647+1.3777\times \log_{e}\left(H\right)$$

$$\log_{e}\mathrm{BGB}=-0.38688+2.0106\times \log_{e}\left(H\right)$$

Aboveground biomass (AGB) and belowground biomass (BGB, in kg) were predicted as a function of tree height (H, in m) (Goodman *et al*., 2013). For herbaceous biomass, we established three 0.25 × 0.25 m subplots located 2 m away from the center of the main plot and collected the total aboveground biomass and the belowground biomass at 0.2 m depth (Fig. S2e). The samples were dried until constant mass and weighed.

### Carbon dating and chemical stability

To estimate how long these ecosystems have been accumulating carbon, we determined the radiocarbon age of the soil and used this to estimate the carbon accumulation rate. We selected four samples from the soil core with the greatest depth in each Vereda to capture the oldest carbon. At each point, the selected samples included the deepest, shallowest, and two intermediates. The analyses were conducted at the Jena C14 laboratory in the Max Planck Institute for Biogeochemistry. Ground, dried samples were combusted using an elemental analyzer‐isotope ratio mass spectrometer (EA‐IRMS), and the resulting CO_2_ was graphitized and analyzed on a Micadas accelerator mass spectrometer (AMS; IonPlus, Switzerland) (Steinhof *et al*., 2017). We converted bulk radiocarbon ages to calendar ages using the OxCal software (Ramsey, 2009) using the calibration curves SHCal20 (Hogg *et al*., 2020) and Bomb 21SH12 (Hua *et al*., 2022), although ages must be viewed as approximations as peat organic matter may not be a completely closed system because soil carbon can be transported. Reported errors reflect combined uncertainties of AMS measures and associated standards (Steinhof, 2013). To obtain the carbon accumulation rate we calculated the net rate of height increment (m yr^−1^) in each layer between two radiocarbon samples and multiplied by the mean carbon density (g m^−1^) in the same layer (Tolonen & Turunen, 1996; Clymo *et al*., 1998). It is important to note that dating bulk peat samples introduces some uncertainty, as organic carbon can be translocated along the soil profile. Ideally, individual macrofossils would be dated to avoid this issue; however, no identifiable macrofossils were available in our samples, with the exception of a single wood fragment.

To assess chemical stability, we employed a method proposed by Hodgkins *et al*. (2018), examining the ratio of labile to recalcitrant compounds. Using this method, we estimated the concentrations of holocellulose (labile) and Lignin (recalcitrant) through basal normalization of infrared spectra (Fig. S3). The compound concentrations can be inferred by analyzing the peaks near the wavenumbers 1030 cm^−1^ and 1510–1630 cm^−1^, respectively. We used the Fourier Transformed Infrared Spectrometry (FT‐IR) Nicolet 6700 at the Calibration and Analytical Resources Laboratory (LRAC), University of Campinas. The samples were prepared in pellets pressed with KBr, in a proportion of 0.05% by weight. Measurements were carried out in transmittance mode using the FT‐IR Imaging Microscope with a resolution of 4 cm^−1^. We processed the spectra and obtained the total holocellulose and lignin concentrations through R packages ‘IR’ (Teickner, 2025) and ‘IRpeat’ (Teickner & Hodgkins, 2022). We used the values previously found by Hodgkins *et al*. (2018) for Tropical and Subtropical Peatlands to compare with the values found for Veredas (see supplementary material in Hodgkins *et al*., 2018).

### Mapping

To estimate the potential relevance of Vereda ecosystems for regional carbon storage, we predicted the total Vereda area across the Cerrado using a machine learning classification algorithm. Since peat occurrence data are not available for the entire biome, we were unable to model peatland extent directly. Instead, we relied on a vegetation cover classification, based on evidence that organic carbon accumulation occurs in Veredas across the Cerrado. It is therefore important to stress that our classification focuses on mapping Vereda ecosystems rather than peat or organic soils.

To perform the land cover classification, we used a combination of remote sensing datasets to train a random forest model. We used Sentinel‐1 (VV and VH backscatter) and Sentinel‐2 (B2, B3, B4, B5, B6, B7, B8, B11, and B12) imagery from 2018 to 2023, grouped into four quarterly periods. We derived the Normalized Difference Vegetation Index (NDVI; Tucker, 1979), the difference between NDVI in dry and wet seasons, the mid‐infrared band ratio (SWIR2/SWIR1; Misra *et al*., 2020), and the polarization ratio (VH/VV; Dubois *et al*., 2020). We also obtained the Digital Elevation Models and the Topographic Wetness Index (Beven & Kirkby, 1979; Riihimäki *et al*., 2021). We used 15 008 reference points as input, including 3208 representing Veredas (including points with or without *M. flexuosa*). The remote sensing imagery was obtained and processed in Google Earth Engine, while the modeling was performed in RStudio (R Core Team, 2025) using the tidymodels (Kuhn & Wickham, 2020), sf (Pebesma, 2018), and terra (Hijmans, 2025) packages. Additional details on the Vereda mapping procedures are provided in the Supporting Information.

### Soil carbon fluxes

To understand how climatic seasonality affected emissions of CO_2_ and CH_4_ in Veredas, we measured the soil fluxes of both gases periodically over a year, sampling during dry (July and September 2023), wet (January 2023 and February 2024), and transition seasons (April and December 2023). Two of our initial sites measured for soil carbon were impacted by fire before measuring the fluxes (ING and RAJ). To avoid the influence of this event on our measurements, we excluded these sites. Because of this loss, we added a new site (CRP, −14.0076, −47.5168) not included in the soil sampling. Thus, we measured fluxes in five Veredas.

In each Vereda, we measured three points inside the waterlogged area (only two in VVT due to its smaller area), totaling 14 sample points across all Veredas (Fig. S2c). To minimize the confounding effects of spatial and temporal variability in water availability, we selected the most waterlogged region during the wet season at each site. We also measured three paired locations (only one in VVT due to a lack of dry areas) external to waterlogged areas to understand the effect of flooding on emissions (Fig. S2c). Measurements were taken using the closed chamber method, with a LI‐COR SmartChamber connected to an LI‐7180 Trace Gas Analyzer. Details on flux measurements are in Supporting Information.

### Environmental variables

To better understand the factors driving the measured fluxes, we also collected data on precipitation, water table depth, soil moisture, and soil temperature. The precipitation data were obtained from a meteorological station installed in a privately owned area near the national park (−4.182°S −47.5715°W). We observed that fluctuations in the water table were not always synchronized with precipitation events. A lag is expected because a minimum rainfall volume is required to recharge groundwater reservoirs and trigger a rise in the water table after the dry season. For this reason, we used two measures: accumulated precipitation over the previous 3 months, which better represents subsurface water availability than monthly precipitation alone, and a temporal lag in precipitation data. We tested the effect of different lag lengths on fluxes, and the final lag was determined based on the model with the highest predictive power (Table S2). Based on this procedure, we used a 2‐month precipitation lag in the subsequent analyses. We installed one piezometer at each flux measurement location inside the flooded areas and collected the data during the flux measurement campaigns. The piezometers were installed deep enough to exceed the minimum water table levels observed during the dry season at each site, with maximum depths of 2 m. Finally, we measured the soil moisture and temperature at 5 cm depth through the dielectric impedance method with a HidraProbe (Seyfried *et al*., 2005).

As commonly found in Veredas, the studied points presented two inundation patterns, with six locations saturated the entire year (ever‐wet) and eight locations seasonally lacking saturation (seasonal). To delimit the two groups, we considered points with surface soil moisture (0–10 cm) < 70% in the dry season (September) as ‘Seasonally Flooded’ and > 70% as ‘Permanently Flooded’.

To assess whether the precipitation pattern in Veredas differs from other tropical peatlands, we calculated the seasonality index for tropical peatlands, including Veredas. We first compiled peat occurrence data from Global Peatland Map 2.0 (Global Peatland Database, 2022) and monthly precipitation from the TerraClimate dataset (Abatzoglou *et al*., 2018). A spatial grid of 5 km was applied and peat occurrence within each grid cell was identified. We also added peat locations from Beer *et al*. (2024) and the sampling sites from this study. In total, we obtained 711 points, of which 180 correspond to Veredas. For each point, we extracted monthly precipitation from 2004 to 2024 using the Qbms package in Rstudio (Al‐Shamaa *et al*., 2025) and calculated the seasonality index following the equation proposed by Walsh & Lawler (1981) below:

$$\mathrm{SI}=\frac{1}{R}\;\sum_{n=1}^{n=12}\left|\underline{x_{n}}-\frac{\underline{R}}{12}\right|$$

where the seasonality index (SI) is measured in function of mean annual precipitation (*R*) and mean month precipitation $\underline{x_{n}}$.

### Data analysis

To interpret the total carbon storage and age, we used mean carbon density, carbon stock, and carbon age values and confidence intervals on a 95% confidence level (*z* = 1.96). To test if ‘carbon storage’ measured using samples with SOC > 8% (the threshold for organic soil in Brazilian soil classification; Santos *et al*., 2018) differed from ‘carbon storage’ including all samples, we ran a *t*‐test. To assess if ‘carbon stability’ differed between the two flooding patterns (seasonally vs permanently flooded), we ran linear mixed‐effect models (LMMs) with ‘flooding pattern’ as a fixed effect, and ‘point’ nested in ‘site’ as a random effect to account for variability among sampling points nested within sites. To compare our results with those from other tropical regions reported by Hodgkins *et al*. (2018), we ran an analysis of variance (ANOVA) with ‘data origin’ (‘Vereda’, *n* = 59, and ‘tropical peatlands’, *n* = 127) as the predictor and ‘compound concentrations’ (‘lignin’ and ‘holocellulose’) as the response variables. This comparison allowed us to test whether lignin and holocellulose concentrations differed between samples collected in Veredas and those from tropical peatlands reported by Hodgkins *et al*. (2018).

In the different models reported here, we selected a mixed‐effects approach (LMM) because points and sites have intrinsic characteristics that influence the response variable but are not the focus of our hypothesis. By including random effects, we allow the model to vary intercepts (and slopes when supported by the data structure), thereby accounting for the hierarchical variability in our data. This approach enables us to estimate the fixed effect without inflating it with variation attributable to differences among random factors.

To understand how the precipitation affects other environmental variables we ran two LMM. In the first, we tested the effect of ‘precipitation’ and ‘flooding pattern’ on ‘water table depth’. We added an interaction factor between ‘precipitation’ and ‘flooding pattern’ as the effect size of ‘precipitation’ on ‘water depth’ is likely different in the two flooding patterns. We also added a quadratic effect of ‘precipitation’ because the relationship presented a non‐linear pattern. ‘Site’ was added as a random effect to address the variability among different sites. In the second, we tested the ‘precipitation’ effect on ‘soil temperature’ with ‘precipitation’ as a fixed effect and ‘point’ nested in ‘site’ as a random effect.

To assess the impact of climatic variability on CO_2_ and CH_4_ fluxes, we ran four LMMs. In all models, fluxes were log‐transformed to meet model assumption of normality and homocedasticity. First, we tested if emissions inside vs outside flooded areas differed across months (January, April, July, September, December, February) using a model with ‘position’ (‘inside flooded area’ and ‘outside flooded area’), ‘month’, and their interaction term as fixed effects (i.e. ‘position*month’), and ‘repetition’ nested in ‘point’ nested in ‘site’ as random effect to address the variability of spatial repetitions, points and sites. Second, we examined if emissions inside flooded areas varied among months in different flooding patterns (seasonally vs permanently flooded) using ‘month’ interacting with ‘flooding pattern’ as fixed effects and the same random effect structure as the previous model. Third, knowing that soil temperature is also a strong predictor of microorganism metabolic activity (Frey *et al*., 2013), we explored if ‘accumulated precipitation’ and ‘soil temperature’ interacting with ‘flooding pattern’, influenced CO_2_ and CH_4_ emissions, also adjusting the same random effect structure described above. For the last, we employed backward selection on the previously described structures to determine the best explanatory models using *P*‐values lower than 0.05 as criteria. To interpret the models' results, we used the *P*‐values; whenever we found a significant result, we calculated the marginal r^2^ (representing how much variance is explained by fixed variables) and conditional r^2^ (representing how much variance is captured by both fixed and random factors) (Nakagawa & Schielzeth, 2013).

All analyses were conducted in R software v.4.3.3 (R Core Team, 2025), utilizing the packages dplyr for data manipulation (Wickham *et al*., 2023), ggplot2 (Wickham, 2016), and sjPlot (Lüdecke, 2023) for visualization, car (Fox & Weisberg, 2019), lsmeans (Lenth, 2016), and performance (Lüdecke *et al*., 2021) for assessing models' significance, explained variations and model assumptions.

## Results

### Veredas environmental conditions

**Fig. 2:**
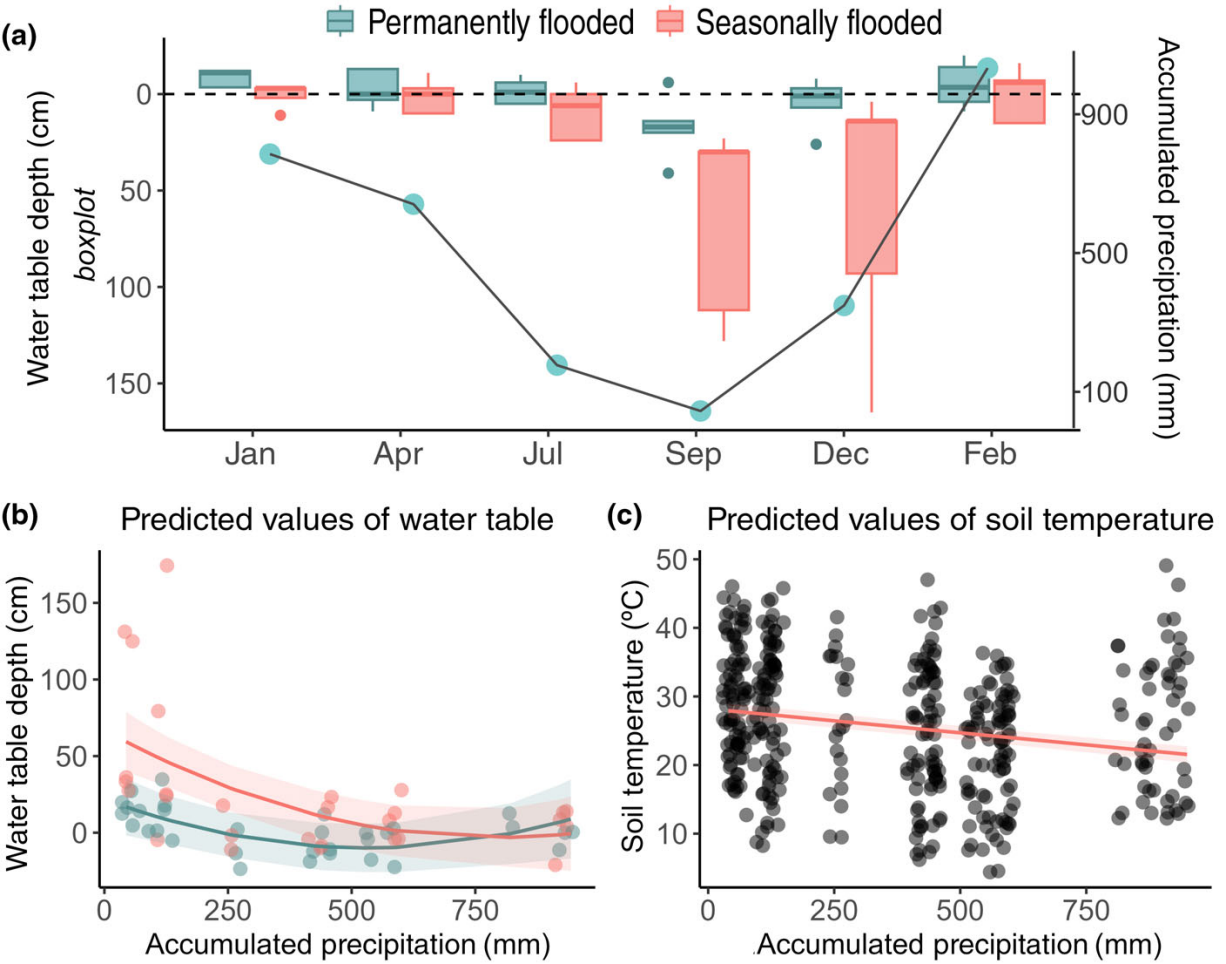
Variation of water table depth, accumulated precipitation, and soil moisture along the different periods in permanently (green) and seasonally (red) flooded Veredas areas. In (a), the box plots represent the water table depth values (cm) sampled at each point. The boxes indicate the interquartile range (first to third quartiles), and the horizontal line within each box represents the median. Negative values represent water above the soil surface. The horizontal dashed line represents 0 cm in the ‘water table depth’ axis. The dots represent the precipitation accumulated in 90 d (mm). (b) Effects of precipitation, flooding pattern, and their interaction term on the water table depth. Note, the quadratic response was significant. (c) Effects of accumulated precipitation on soil temperature. Lines and shades in (b) and (c) show predicted probabilities and 95% CIs, respectively, while points show raw data.

The annual precipitation during the sampling period was 1321 mm, with 92% occurring during the wet season. The mean water table depth during dry months was 22 cm (±38 SD; July, September and December) and −2 cm (±10 SD) during wet months (January, February and April), with negative water table depth values indicating water above the soil surface. The water table depth in Veredas was highly correlated with accumulated precipitation (*P* < 0.01, Fig. 2a). Water table response to precipitation, however, changed between permanently and seasonally flooded points (Fig. 2b). Accumulated precipitation also affected soil temperature (*P* < 0.001, Fig. 2c).

**Fig. 3:**
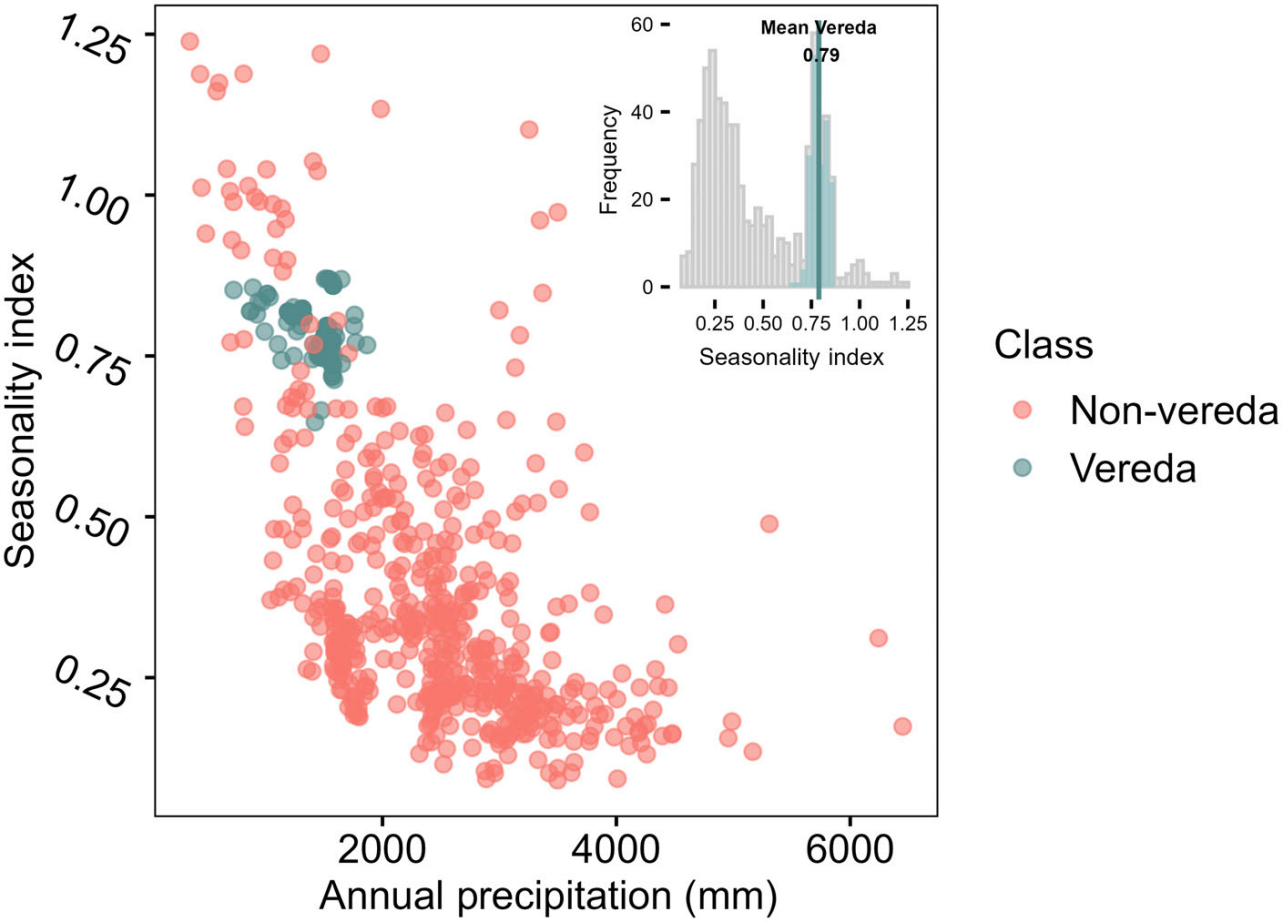
Seasonality index and mean annual precipitation between 2004 and 2024 for tropical peatlands. Seasonality index was measured following Walsh & Lawler (1981). Precipitation data were obtained from Terra Climate dataset. Green points represent Veredas sites and pink represent other peatland ecosystems. The top right inset shows the histogram of seasonality index values with Veredas values highlighted in light green. The average seasonality index for Veredas, 0.79, is highlighted in a vertical line.

Veredas exhibited greater climatic seasonality than other tropical peatlands with a mean SI of 0.79 (±0.01), compared to 0.37 (±0.02) for non‐Veredas (Fig. 3). In addition to higher seasonality, Veredas received lower annual precipitation than the non‐Veredas average, with a mean of 1416 mm (±27 mm) per year, whereas other sites receive on average 2446 mm (±77 mm; Fig. 3). According to Walsh & Lawler (1981), sites with a SI higher than 0.6 are classified as seasonal, and those exceeding 0.8 as markedly seasonal with a long dry season. Only 13% of non‐Veredas sites sampled here presented a SI above 0.6. On average, Veredas were located in the 83^th^ percentile of the distribution for the SI.

### Carbon stocks in Veredas

**Fig. 4:**
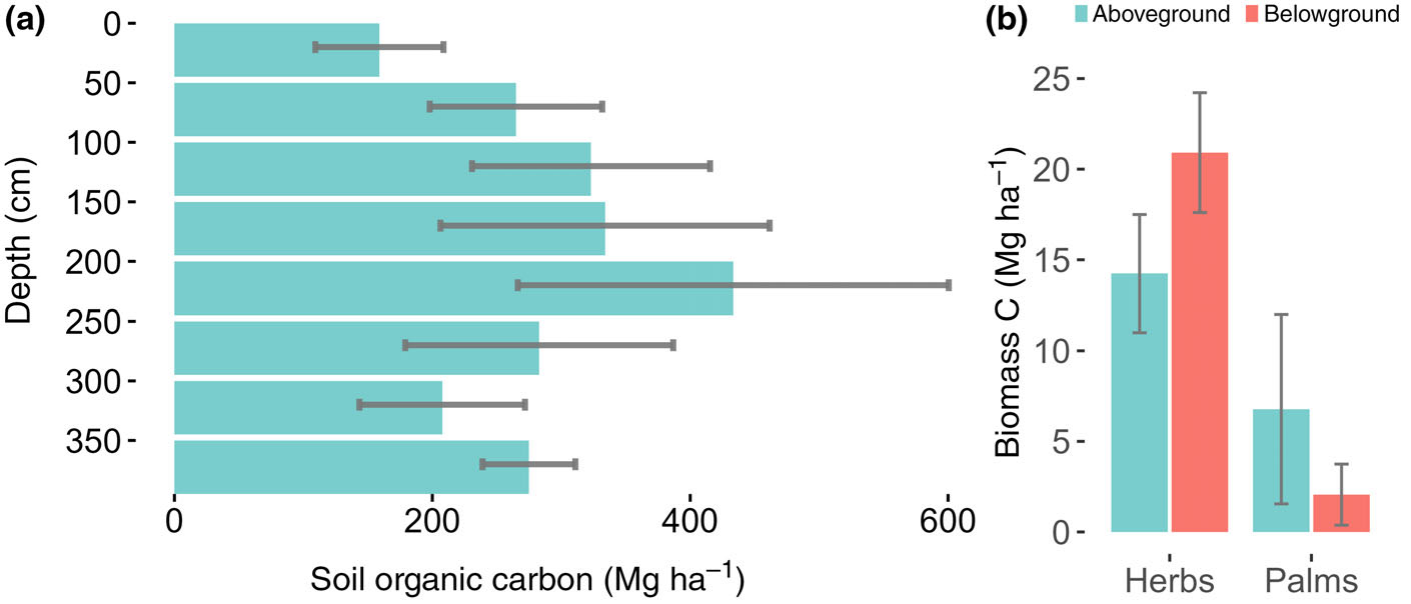
Total SOC and biomass C of the studied wetlands. (a) Mean variation in soil carbon stock with depth per hectare (or C density) averaged across the six studied sites. The error bars represent the 95% CIs. (b) Variation in biomass C for herbs and palms. Aboveground C is represented by green bars, while belowground C is shown in red. Error bars indicate the 95% CIs.

Across all sites, the SOC density in Veredas was 1159 MgC ha^−1^ ± 284 (Fig. 4a). This stock was distributed in organic soils with a mean thickness of 1.9 m ± 0.2 (Table 1), soil dry bulk density of 759 kg m^−3^ ± 60 (Table 1), and 8.9% ± 1.0 of carbon (Table 1). Carbon ranged from 0.3 to 32%. Total area of the Veredas where we estimated total carbon stocks ranged from 0.4 to 28 ha. Higher carbon concentrations were found between 50 cm and 2 m, with relatively lower carbon in the first centimeters (Fig. 4a). In three of the six sites, we sampled at least one point with organic soil up to 4 m in depth (Fig. S4). We did not find differences between permanently and seasonally flooded Veredas.

**Table 1:** Mean (±95% CIs) of parameters used to calculate the total carbon stock in each Vereda.

Site	Coordinates	No. of cores	Mean depth (m)	Mean C (%)	Dry bulk density (kg m^−3^)	Area (ha)	C density (Mg ha^−1^)	Max C density (Mg ha^−1^)	Min C density (Mg ha^−1^)	Flooding pattern
BBT	−14.0984 −47.6476	9	2.13 (±0.3)	8.9 (±1.8)	672 (±135)	3.98	1265 (±255)	1740	458	Permanently and seasonally
ENG*	−13.6231 −47.4760	9	1.65 (±0.74)	5.1 (±2.3)	1094 (±150)	3.38	663 (±353)	1533	89	Permanently
FDS	−14.1906 −47.5782	9	2.28 (±0.59)	7.5 (±1.8)	857 (±121)	4.06	1440 (±522)	2498	173	Permanently and seasonally
ING*	−14.1417 −47.7510	10	2.28 (±0.61)	9.3 (±2.4)	640 (±127)	16.94	1243 (±384)	2374	323	Seasonally
RAJ*	−14.1424 −47.7067	11	1.77 (±0.58)	11.2 (±2.7)	696 (±139)	28.69	1512 (±589)	3684	157	Permanently
VVT	−14.1816 −47.5715	6	0.93 (±0.28)	10.8 (±4.8)	650 (±213)	0.39	717 (±557)	1854	194	Seasonally

Mean and CIs were calculated for mean thickness and carbon density at the point level, and for mean C and dry bulk density at the sample level. Data for area, maximum C, and minimum C density are absolute values. Sites with sample depths up to 4 m were marked with an asterisk. C, carbon; max, maximum; min, minimum.

Considering just the samples with carbon > 8%, we obtained a carbon density of 969 MgC ha^−1^ (± 248, Table S5; Fig. S5), with a mean total depth of 155 cm (±28, Table S5; Fig. S5). Note that this is the sum of layers with carbon > 8%, not necessarily subsequent layers. Moreover, mean carbon content was 16% (± 1%, Table S5) and mean soil bulk density was 636 kg m^−3^ (±78, Table S5). Since there were no significant differences in samples with or without carbon < 8% (*t* = 0.94, *P* = 0.36), we used the dataset including all samples.

The total palm biomass in Vereda sites was 18.8 Mg ha^−1^ (±14.7), with 23% of biomass belowground (Fig. 4b). The total herbaceous biomass was 66.2 Mg ha^−1^ (±10.6), with 67% of this amount contributed by belowground biomass in the first 20 cm (Fig. 4b). Considering a conversion ratio of 0.47 for biomass to carbon (IPCC, 2023) we obtained a total mean carbon stock in Veredas of 1199 Mg ha^−1^ (±296, Fig. 4). In this total storage, 96% was from SOC and just 4% was carbon stored in below‐ and aboveground living biomass.

### Carbon age and stability

**Fig. 5:**
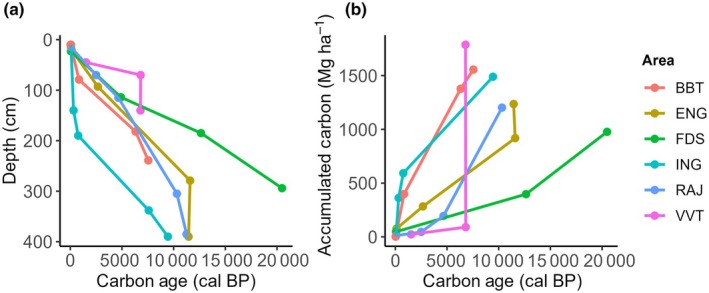
Calibrated carbon age obtained through radiocarbon dating for six Veredas along the soil profile. (a) Carbon age at different depths and (b) the cumulative amount of carbon as a function of time.

Basal ages for organic soil ranged from 7547 to 20 481 yr calBP across the sites, with a mean of 11 185 yr calBP (±5185). The maximum age was found at 294 cm depth in the FDS site (Table 2; Fig. 5a). While the oldest was not the deepest, there are possible older ages for the areas where the sampling stopped at 4 m (Fig. 5a). At the ING site, we observed a clear stratification in carbon age: recent carbon (from the past millennium) was found in the upper 2 m, while deeper layers (> 2 m) contained much older carbon (older than 1000 yr, Fig. 5a). By contrast, we did not observe a difference in ages for the two deepest samples at VVT and ENG (Fig. 5a). We also sampled a well‐conserved piece of wood at 338 cm in the ING site, which dated to 7590 yr calBP, further supporting the presence of ancient organic material. Rates of carbon accumulation varied in the past for many of the sites (Fig. 5b). Overall, the slowest rates of C accumulation were in the oldest area, which is also the richest in carbon (FDS site, Fig. 5b; Table 1). It is important to note that carbon‐density values reported in Table 1 are based on nine cores in each Vereda, while the values used in Fig. 5 are derived from the single core where radiocarbon dating was performed. Discrepancies between these two datasets may reflect differences in sampling resolution. The mean carbon accumulation rate for the measured layers within the six sites was 25.6 gC m^−2^ yr (±16.3).

**Table 2:** Age and chemical stability data for each Vereda.

Site	Min F14C	Max calendar age range (calBP)	Max probability age calibration (%)	Mean holocellulose (%)	Mean lignin (%)
BBT	0.4373 (0.0013)	7501–7593	67.2	21.1 (7.8)	11.6 (1.8)
ENG	0.2862 (0.001)	11 290–11 597	68.2	17.7 (5.3)	10.8 (1.2)
FDS	0.1215 (0.0008)	20 345–20 618	95.4	24.5 (1.2)	16.6 (6.9)
ING*	0.3526 (0.0011)	9346–9540	93.3	22.6 (1.3)	16.8 (3.6)
RAJ*	0.3211 (0.001)	10 262–10 368	93.8	17.0 (6.4)	13.0 (2.2)
VVT	0.4761 (0.0013)	6738–6873	88	11.4 (3.1)	11.8 (2)

F14C represents the fraction of modern carbon, while Calendar Age denotes the calibrated carbon age determined using curves SHCal13 and Bomb 21SH12. Both values were derived from the deepest sample at each point, with error values in parentheses, obtained from radiocarbon analysis. Sites with sample depths up to 4 m are marked with an asterisk. Max Probability Age Calibration indicates the probability that F14 data align with the Calendar Age calculated using OxCal software, with all calendar ages representing the values with the highest probability. Mean Holocellulose and Mean Lignin represent chemical stability data obtained through infrared spectra and basal normalization, following the method proposed by Hodgkins *et al*. (2018). For Mean Holocellulose and Mean Lignin, the reported values represent means and SD in parentheses.

**Fig. 6:**
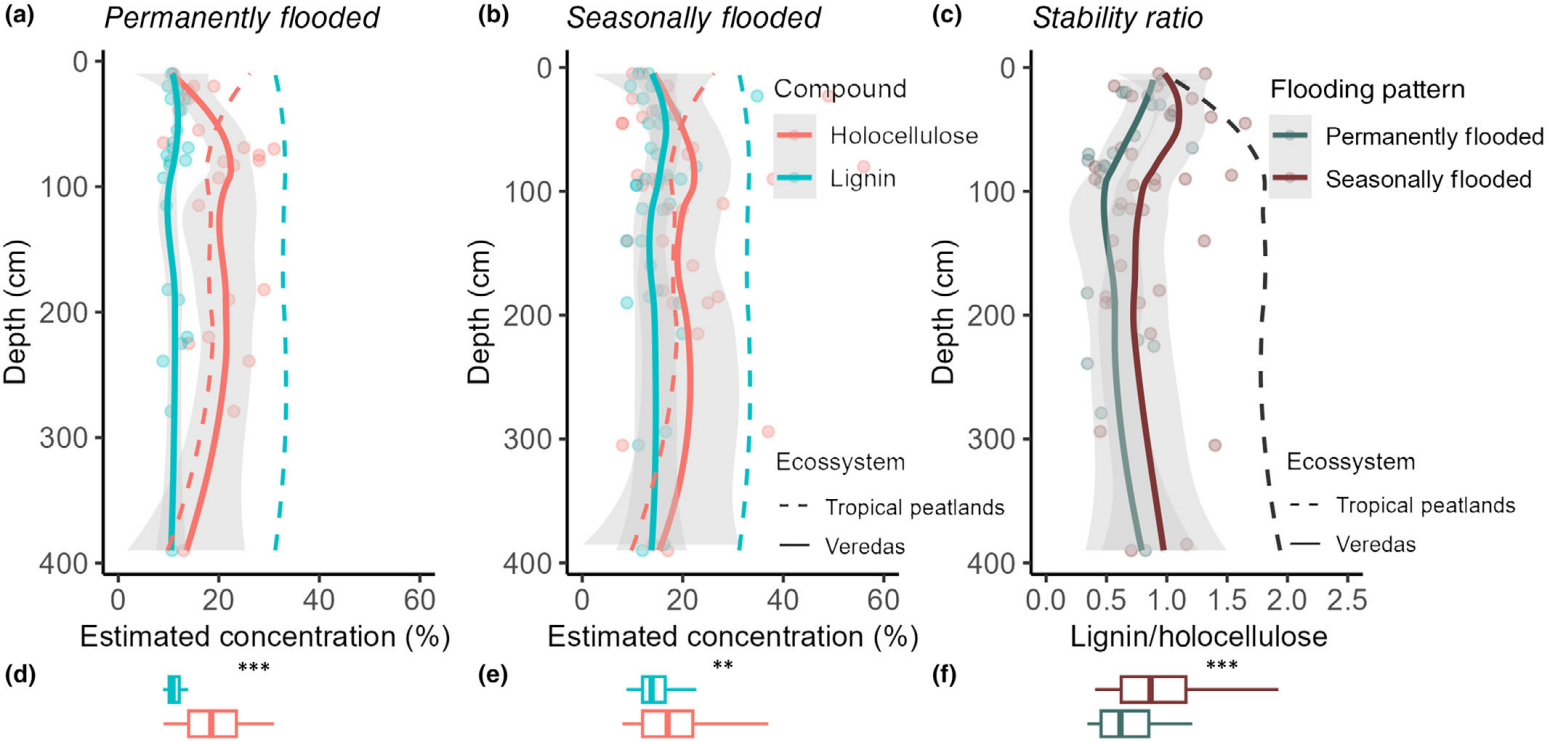
Estimate of carbon stability through the proportion of holocellulose in red (labile compound) and lignin in green (recalcitrant compound) for the sample within six Veredas. Higher concentrations of recalcitrant compounds indicate greater stability of peat to decomposition; conversely, higher concentrations of labile compounds indicate lower stability. Our data (continuous line) can be compared with the average for tropical peat from Holdkgins *et al*. (2018; dashed lines). Compound proportions for sites (a) flooded year‐round across the soil profile, and (b) flooded only during the wet season across the soil profile. (c) The ratio between lignin and holocellulose. In (a), (b), and (c), data were summarized using locally weighted polynomial regression (LOESS) smooth curves, with shades indicating 95% CIs derived from sampling variability. Note that the tropical peat from Holdkgins *et al*. (2018) is the same in (a) and (b). (d), (e), and (f) Box plots for Veredas data exhibiting differences in linear mixed‐effect models, highlighted by asterisks, with ***, *P* < 0.001 and **, *P* < 0.01. The boxes indicate the interquartile range (first to third quartiles), and the horizontal line within each box represents the median. For them, *x*‐axes are the same as in (a,b,c).

For the chemical resistance to decomposition, we found a distinct pattern in stability ratios between permanently vs seasonally flooded points, with a larger difference between lignin and holocellulose on the permanently (Fig. 6c, estimate = 0.08, *P* < 0.0001) than the seasonally flooded Veredas (Fig. 6d; estimate = 0.04, *P* = 0.0027). The lignin (*t* = 2.242, *P* = 0.02) but not the holocellulose (*t* = −0.214, *P* = 0.83) content was significantly different between the two types of Veredas. The mean concentration of lignin was 13.4% (±1.3 SD), and holocellulose was 19.2% (±2.5 SD). For permanently flooded sites, the mean lignin was 11.1% (±0.6 SD), and the mean holocellulose was 19.1% (±2.8 SD), while for seasonally flooded sites, the mean lignin was 14.9% (±1.6 SD) and the mean holocellulose was 19 (±3.8 SD). Furthermore, Veredas had less lignin, the more recalcitrant compound, than the average from other tropical peats (Fig. 6a,b; *F* = 1888.5, *P* < 0.0001). However, they had similar amounts of holocellulose (Fig. 6a,b; *F* = 0.079, *P* = 0.78). The ratio between lignin and holocellulose was more than two times higher in other tropical peatlands than in Veredas environments (Fig. 6f).

### Veredas predicted distribution

**Fig. 7:**
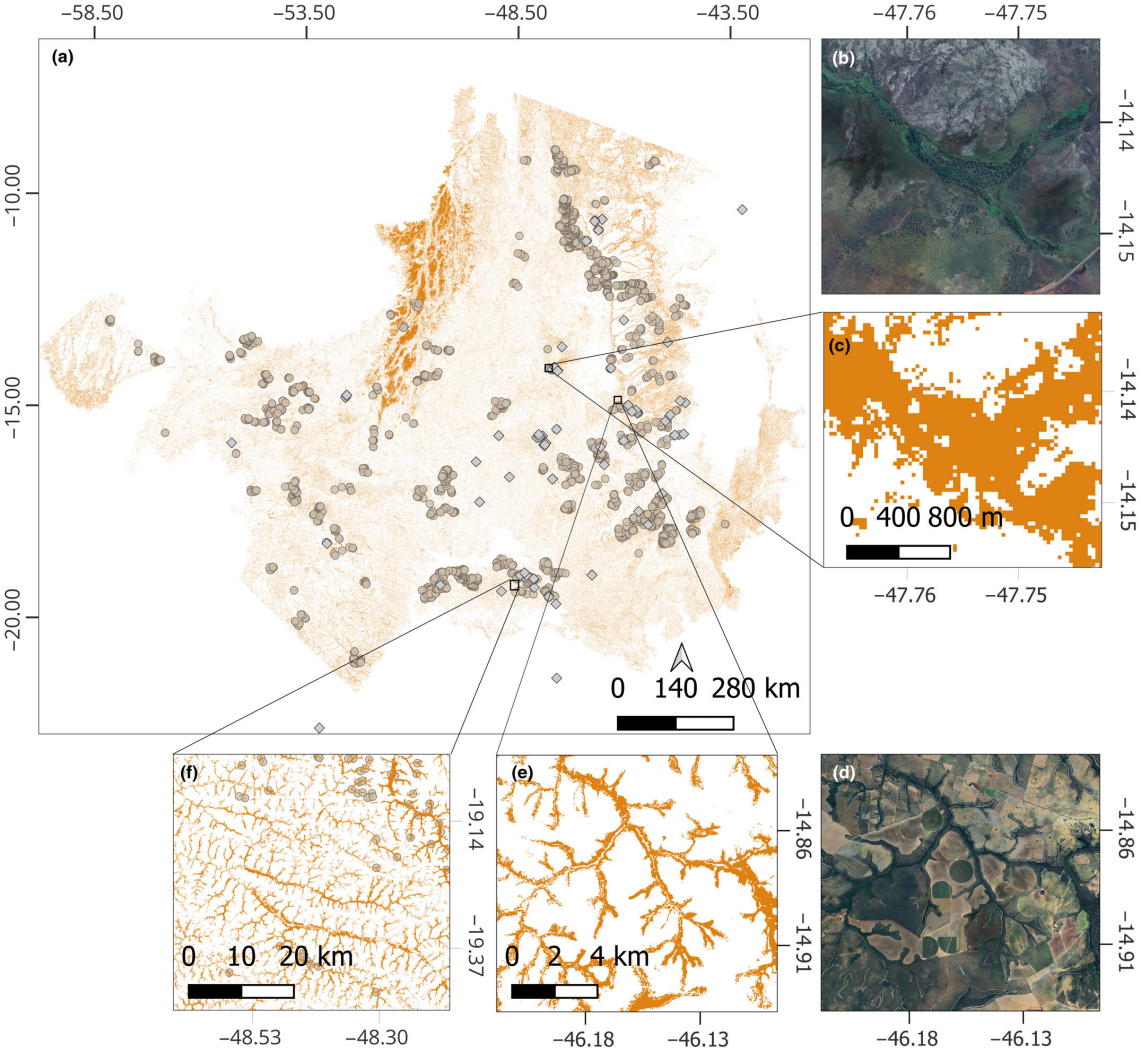
Wetland area predicted in Cerrado. The light orange points represent the training points for Veredas obtained through SICAR. The gray diamonds represent 171 peat occurrence verification points from Beer *et al*. (2024) and our sites. We limited the predicted area to regions where we have training points. The prediction was obtained through a random forest model, trained with spectral indices from Sentinel 1 and 2 and a digital elevation model. (a) the entire predicted area. (b, c) *c*. 350‐fold magnification of the ING site with prediction and RGB images, respectively. (d, e) *c*. 70‐fold magnification in a landscape of a mixed matrix of agriculture and native vegetation. (f) *c*. 14‐fold magnification of the Veredas, highlighting the branched patterns.

Remote sensing initial classification predicted *c*. 19 Mha of Veredas. After correction using precision values (commission error), the predicted Vereda area was 16.7 (±1.2) Mha, representing *c*. 8% of the entire domain and 2% of the entire Brazil (Fig. 7; Table 3). The classification was run 10 times and achieved mean overall accuracy of 82% (±2.4), with 86.4% (±7.2) of precision and 80.5% (±5.9) of recall for the Veredas class (Tables 3, S6). The mean omission error, measured by recall values, was greater than commission, indicating a possible higher underestimation than overestimation of the Veredas class. Our map explicitly incorporated the 144 ground‐truth points of peat occurrence, derived from previous records (Beer *et al*., 2024) and our own sampling.

**Table 3:** Mean performance metrics for the classification model (confidence intervals in parentheses), considering all classes and Veredas individually.

Class	Accuracy (%)	Precision (%)	Recall (%)	F1 Score (%)	Predicted area (Mha)
All classes	82.1 (±2.4)	–	–	–	
Veredas	95. 6 (±6.4)	86.4 (±7.2)	80.5 (±5.9)	83 (±5.2)	16.7 (±1.2)

### Carbon fluxes

The mean rate of CO_2_ efflux across the six sampled Veredas was 1430 gC m^−2^ yr (±257) while the mean CH_4_ efflux was 3.0 gC m^−2^ yr (±0.4). The maximum CO_2_ emission measured was 22 150 gC m^−2^ yr in a seasonally flooded point in September, and the maximum CH_4_ emission was 40 gC m^−2^ yr in a permanently flooded Vereda in December. Seventy percent of the total emissions occurred during dry months.

**Fig. 8:**
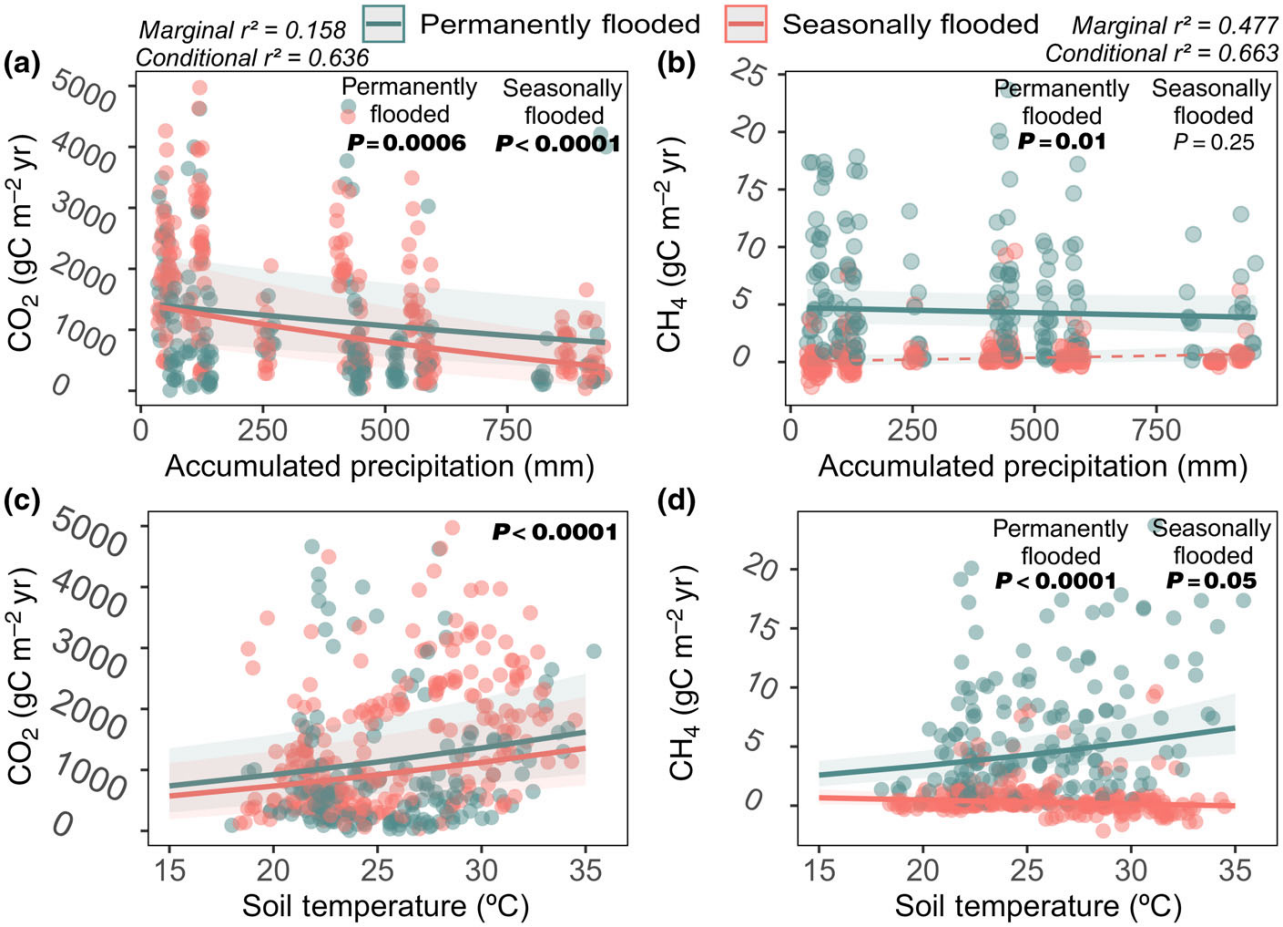
Carbon emissions in response to accumulated precipitation and soil temperature. Green lines and points represent ‘permanently flooded’, and red lines and points represent ‘Seasonally Flooded’ Veredas. Shades represent SE. Marginal *r* ^2^ for the model with CO_2_ is 0.158 and conditional *r* ^2^ is 0.636. For CH_4_ model, marginal *r* ^2^ = 0.477 and conditional *r* ^2^ = 0.663. (a, b) Show the effect of ‘accumulated precipitation’ on CO_2_ or CH_4_ emissions. Models *P*‐values for the interaction between flooding patterns and precipitation are reported in the plot. (c, d) Show the effect of soil temperature on CO_2_ or CH_4_ emission. In (c), only a unique *P*‐value was presented because the interaction between soil temperature and flooding pattern was not included in the model after backward selection. Both lines present the same slope and intercept, and the reported p‐value is the overall effect of soil temperature on CO_2_ emission. In (d), the interaction effect was still present after backward selection; both *P*‐values are presented.

Emissions of CO_2_ differed inside and outside the flooded areas (Fig. S6a; Table S7), with dry soils emitting 31% more than flooded ones (Fig. S7). For CO_2_ emissions inside the flooded area (i.e. the actual Veredas emission), the effect of time (accessed through the difference between months) and precipitation was stronger in seasonally flooded Veredas (Figs 8a, S8a,b, S9a; Tables S7, S8). We found a strong effect of month with greater CO_2_ values in September and December (Figs S8a,b; S9a) and a marginal effect of the interaction between months and flooding pattern (Veredas seasonally or permanently flooded; Table S7; Fig. S9a). Climate seasonality significantly affected fluxes through accumulated precipitation (Fig. 8a; Table S8), soil temperature (Fig. 8c; Table S8), and interaction between accumulated precipitation and pattern in seasonally flooded Veredas (Fig. 8a; Table S8).

For CH_4_, we also found differences in emissions inside and outside the flooded areas (Figs S6b, S7b; Table S7), with an estimated value 20 times higher inside than outside (Fig. S7b). However, distinct from CO_2_, this pattern was consistent for the entire year and always higher inside the flooded area (Fig. S6b).

For CH_4_, we detected a significant effect of precipitation only in permanently flooded Veredas (Fig. 8b; Table S8). Emissions were only different in September, at the peak of the dry season, being greater than other months (Figs S8c,d, S9a). Permanently flooded Veredas exhibited substantially higher CH_4_ emissions, exceeding the seasonal average by more than tenfold. By contrast, seasonally flooded Veredas acted as methane sinks during dry months (Figs S8c,d, S9a; Table S7). This divergence was further amplified by temperature responses: while CH_4_ emissions increased with temperature in permanently flooded areas, a negative relationship was observed in seasonally flooded Veredas (Fig. 8d; Table S8).

## Discussion

Our findings highlight the significant carbon storage potential of open wetlands in Cerrado, driven primarily by the accumulation of peat and organic soils. These carbon stocks have accumulated over the last 20 000 yr, indicating that despite pronounced seasonal fluctuation in hydrological conditions, many Veredas have maintained sufficiently stable waterlogged (anoxic) conditions to support long‐term peat formation and persistence. However, the lower lignin content compared to other tropical peat ecosystems suggests that this carbon is chemically less stable and potentially more vulnerable to changing hydrologic conditions. Declines in precipitation and elevated soil temperatures during the dry season increased carbon exposure to aerobic decomposition, resulting in higher GHG emissions during this period. These findings underscore that this critical yet underrecognized carbon pool is highly sensitive to anthropogenic pressures, particularly those that alter the hydrological regime and reduce the duration or extent of waterlogged conditions in the Cerrado.

### High carbon stocks in Cerrado Veredas

Our study is the first to assess carbon stocks in Veredas across multiple sites using deep soil profiles, including layers exceeding 1.6 m. Most previous assessments sampled only superficial layers (e.g. 20–100 cm), which resulted in major underestimation of carbon horizon, or were restricted to a single site (Table 4). Based on our measurements, restricting sampling to these shallow layers would underestimate total carbon by 55–95%. We also detected substantial variation across sites (SD = 32%), highlighting the heterogeneity of carbon accumulation in Veredas and the importance of expanding sampling both geographically and in depth. In a recent perspective article about current knowledge about Cerrado peatlands, Beer *et al*. (2024) also highlight the underrepresentation of these ecosystems; among 171 peat locations reported across 34 studies, only two included measurements of carbon stocks at depths > 1 m. Some previous studies adopted a fixed sampling depth rather than measuring the full soil profile. Examining diverse Vereda sites with distinct accumulation patterns is crucial for accurately assessing the regional carbon budget of these ecosystems.

**Table 4:** Carbon density measured in Veredas by previous studies.

Paper	*n*	Depth (m)	Storage (MgC ha^−1^)	Storage cm^−1^ (MgC ha^−1^)
Wantzen *et al*. (2012)	3	0.6	125.0 (±75)	2.1 (±1.2)
Sales *et al*. (2020)	1	1.0	125.0	1.2
Soares *et al*. (2021)	3	0.3	140.6 (±93)	4.7 (±3.1)
Horák‐Terra *et al*. (2022a)	1	1.5	334.1	2.2
Horák‐Terra *et al*. (2022b)	1	1.6	532.5	3.3
Horák‐Terra *et al*. (2022b)	1	1.2	331.1	2.7
Santos *et al*. (2023)	1	1.0	404.6 (±46.5)	4.0 (±0.5)
Santos *et al*. (2023)	1	1.0	576.2 (±67.6)	5.7 (±0.7)
Souza *et al*. (2025)	1	1.2	462.6 (±27.7)	3.8 (±0.2)
Present study	6	4.0	1159.0 (±284)	2.9 (±0.7)

The locations of each study are shown in Fig. 1(c). Note the two rows for Horák‐Terra *et al*. (2022b) and Santos *et al*. (2023), where the authors sampled two cores in the same Vereda. *n* is the number of sampled Veredas. When *n* is > 1, depth values represent the maximum depth. The values in parentheses in the Storage column represent the Standard Deviation, when it was reported.

Even with methodological differences among studies, our mean carbon densities align with values reported elsewhere in the Cerrado (Table 4), reinforcing that high soil carbon storage is a general feature of Veredas. Whereas the combined soil and biomass carbon stocks of dry Cerrado vegetation types rarely exceed *c*. 300 Mg C ha^−1^ (Batlle‐Bayer *et al*., 2010; Miranda *et al*., 2016; Morais *et al*., 2020), our estimates show that Veredas store nearly four times more carbon on average. Importantly, carbon stocks in Cerrado open wetlands are approximately eight times greater than the mean aboveground live biomass carbon of lowland Amazon forests (Saatchi *et al*., 2007). These findings confirm that Veredas represent one of the most important carbon reservoirs not only within the Cerrado, but also at the national scale. This contrast highlights the limitations of forest‐centric carbon accounting approaches, which tend to systematically overlook carbon‐dense, non‐forest ecosystems.

To date, the spatially discontinuous distribution of Veredas, together with their occurrence in a highly seasonal climate, has limited their representation in global carbon assessments and modeling efforts. Although carbon assessments in Veredas remain scarce compared to other peatlands, evidence presented here indicates that Veredas deserve similar consideration to other tropical peatlands world‐wide. The carbon density found here surpasses that reported for Amazonian peatlands in Peru (892 ± 535 MgC ha^−1^; Draper *et al*., 2014), is comparable to Indonesian peatlands (1200–1300 MgC ha^−1^; Page *et al*., 2006; Warren *et al*., 2017), and is close to values reported for the Congo basin peatlands (2186 MgC ha^−1^; Dargie *et al*., 2019). Additional sampling across different regions of the Cerrado, including Veredas in degraded landscapes using a standardized methodology, is required to corroborate this comparison fully.

While the soil carbon content found here (*c*. 9%) is below some thresholds commonly used for peat classification (e.g. 30%), these criteria are not universally agreed upon (Lourenco *et al*., 2022). The Global Peatlands Assessment (2022) applies a 12% threshold, while the Brazilian Soil Classification uses 8% for organic soils. Veredas are among the most seasonal tropical peatlands, with very few ecosystems under similarly seasonal precipitation regimes capable of accumulating peat. Some sites with comparable climatic seasonality, such as the Barotse Floodplain in Africa, maintain peat formation under fluvial flooding or lacustrine water regimes (Global Peatlands Initiative, 2022), whereas Veredas are distinguished by being groundwater‐fed. This hydrological regime, combined with the spatially disconnected distribution of Veredas across the landscape, likely slows carbon accumulation while enhancing organic matter decomposition, resulting in high spatial heterogeneity. Although our samples show comparatively lower carbon content than other areas, they indicate substantial carbon accumulation over millennia (> 5000 yr), high carbon density, and ecosystem characteristics typical of peatlands, such as frequent flooding. Moreover, previous studies reported data from Veredas with even higher carbon content than the one found in our samples, reinforcing the potential of Veredas to function similarly to other peatlands (Beer *et al*., 2024).

### Ancient but less stable carbon in Veredas

We found carbon older than 20 000 yr, suggesting the long‐term environmental stability of this ecosystem. Earlier studies report even older ages (30000–35 000 yr), supporting persistent carbon accumulation through past climatic fluctuations (Ferraz‐Vicentini & Salgado‐Labouriau, 1996; Barberi *et al*., 2000; Horák‐Terra *et al*., 2022a). Palynological analyses suggest that despite climatic fluctuations, Veredas have sustained a moist ecosystem for extended periods, allowing persistent long‐term carbon storage (Horák‐Terra *et al*., 2022a). The accumulation rates we observed are comparable to African peatlands but lower than those from Amazonia and Asia (Dargie *et al*., 2019; Lähteenoja *et al*., 2012; Page *et al*., 2004), likely reflecting drier conditions and lower productivity associated with the Cerrado.

Despite being as old as other tropical peatlands, the carbon in Veredas is not as chemically stable. In a global analysis, Hodgkins *et al*. (2018) and Verbeke *et al*. (2022) found more stable and fewer unstable compounds in tropical than in non‐tropical peatlands. However, when comparing Vereda soil with other tropical regions, we found *c*. 60% fewer stable compounds (lignin) than the tropical average (Hodgkins *et al*., 2018). This is likely due to the different vegetation types forming the organic matter. Amazonian, Indonesian, and Congo peatlands are wet forest environments (Page *et al*., 2006; Draper *et al*., 2014; Dargie *et al*., 2019), while Veredas are an herbaceous ecosystem with a low density of palms, typical of savanna ecosystems (Ribeiro & Walter, 1998). Wood contains a high concentration of lignin (a stable compound), making organic matter in forest ecosystems more resistant to decomposition. Previous evidence suggests that soil carbon derived from C4 plants has faster rates of decomposition than C3‐derived plants (Wynn & Bird, 2007), suggesting that an ecosystem dominated by grasses (C4) is more unstable. Furthermore, despite the low amount of lignin, we expect high amounts of holocellulose. Veredas exhibit similar levels of labile compounds (holocellulose) as other tropical peatlands, suggesting higher decomposition rates in seasonal peatlands as compared to other tropical peatlands dominated by lignified plants. It is interesting to note the lower holocellulose concentrations in the upper peat layers of Veredas, which may reflect intensified decomposition in recent years due to increasing drought intensity (Hofmann *et al*., 2021, 2023, 2025) and is consistent with the lower carbon content observed in surface soil. Thus, despite having a similar potential for carbon storage as other tropical peatlands, Veredas may be much more vulnerable to changes in flooding dynamics caused by climate or land use changes.

We also found lower lignin‐to‐holocellulose ratios in permanently flooded sites compared to seasonally flooded ones, implying that constantly saturated Veredas may be even more susceptible to disturbance if hydrological stability weakens. Because they lack the buffering effect of seasonal fluctuations, shifts in precipitation or groundwater use could rapidly destabilize these long‐standing carbon stocks.

### Veredas are spread throughout the Cerrado

We demonstrate that Veredas ecosystems are distributed across the entire Cerrado, occupying 8% of the total domain area. Beer *et al*. (2024) synthesized current knowledge on peatland distribution in the Cerrado by integrating the Brazilian Soil Map (IBGE, 2021) with the Global Peatland Map (Global Peatland Database, 2022). They highlighted the limitations of existing maps, noting that only 12% of the 171 peatland sites identified in the literature were captured by this combined dataset. Of this, 98% were Veredas and campos úmidos sites, reinforcing that they are the most important peat‐accumulating ecosystem in Cerrado. This result underscores the importance of improving our ability to predict the distribution of Veredas; with more research, we may find a far more extensive, yet largely undocumented, presence of peat and organic soils in the Cerrado. Our predictions suggest that Veredas occupy an area 6× greater than what has been previously mapped as peatlands (Beer *et al*., 2024), revealing a substantial knowledge gap.

In this study, we mapped the extent of Vereda vegetation type rather than directly predicting SOC extension. However, there is evidence of high carbon stored in Veredas soils across all regions of the Cerrado (Beer *et al*., 2024; Fig. 1; Table 4). If the total Veredas area in Brazil has the potential to accumulate carbon as peat, it could represent up to 1.6 PgC (±0.8 PgC; one pentagram is equivalent to one billion metric tons) in the national carbon stock, considering only the first 30 cm of the soil. These estimations consider the minimum depth assessed in previous studies and are based on the average carbon densities previously and here reported (Table 2). Moreover, using the mean value obtained here for soil profiles up to 4 m depth, the total Veredas carbon stock could reach 20 PgC (±1.6 PgC), representing > 20% of the estimated carbon storage of the upland Amazon rainforest (*c*. 86 PgC; Saatchi *et al*., 2007). However, these numbers are uncertain because the proportion of Veredas that are actively forming peat remains unknown.

To contextualize Veredas based on other systems, Winton *et al*. (2025) showed that Colombian palm swamps, ecosystems with many similarities to Veredas, are the most important peat‐accumulating vegetation in Colombia, yet also exhibit the highest variability in soil carbon content. Given the high topographic variability and elevated precipitation seasonality observed in Veredas, we also expect substantial variability in soil carbon content across sites. Furthermore, seasonally or permanently flooded Veredas will likely present different patterns of carbon accumulation that were not captured in our sampling. Understanding the temporal and spatial extent to which these Veredas remain flooded is essential for accurately assessing their influence on carbon dynamics. Together, these findings highlight the need for further research to assess the macrogeographical patterns of peat formation and the variability of carbon stocks under different environmental conditions within Veredas. It remains critical to determine whether, and to what extent, these ecosystems are actively accumulating carbon as peat.

### Large carbon emissions in the dry season

GHG emissions from Veredas soils are relatively high on local landscapes (Mander *et al*., 2025), with a mean CO_2_ flux four times higher than that from other Cerrado vegetation types (Neto *et al*., 2011). Remarkably, mean CO_2_ emissions in Veredas are comparable to some of the highest values reported for American and Asian tropical peatlands, and the maximum emissions we measured are up to 10 times greater than the maximums documented in those ecosystems (Jauhiainen *et al*., 2005; Melling *et al*., 2005; Sjögersten *et al*., 2011; Wright *et al*., 2011, 2013; Hoyt *et al*., 2019; Pärn *et al*., 2023). However, mean CH_4_ emissions are three to four times lower than those from *M. flexuosa* palm swamps in the Amazon, and our maximum values are approximately half of their reported peaks (Teh *et al*., 2017; Pärn *et al*., 2023).

These emissions occur predominantly in the dry season, with *c*. 70% of total flux concentrated in this period, mainly due to high CO_2_ efflux. This pattern reflects climatic seasonality across Cerrado vegetation types, where ecosystems can briefly shift from carbon sinks to sources during drought (Miranda *et al*., 1997; Meirelles *et al*., 2015; Zanella De Arruda *et al*., 2016). While non‐flooded ecosystems experience reduced respiration during drought due to metabolic constraints (Neto *et al*., 2011; Bustamante *et al*., 2012), lower water tables in Veredas increase oxygen availability, enhancing decomposition and amplifying dry‐season GHG emissions. As the Cerrado becomes hotter and drier (Hofmann *et al*., 2021, 2023, 2025), these emissions may intensify.

CO_2_ flux varies strongly across the year in both seasonally and permanently flooded Veredas, but precipitation effects are stronger in seasonally flooded areas, likely due to greater fluctuations in water‐table depth (see Fig. S2). In permanently flooded Veredas, soil temperature plays a larger role in controlling emissions, as oxygen levels remain consistently low. During the dry season, higher temperatures promote microbial respiration (Mander *et al*., 2025), driving seasonal contrasts in emissions.

CH_4_ emissions are less seasonally variable than CO_2_, but they strongly reflect hydrology, with up to tenfold higher CH_4_ emissions in permanently flooded sites, whereas seasonally flooded Veredas can become CH_4_ sinks during dry months. The two flooding systems also have opposite emissions responses to increasing temperature, which are likely due to reduced soil moisture in seasonally flooded Veredas limiting methanogenesis during the dry season, while saturated soils in permanently flooded Veredas maintain active methanogenic communities (Mander *et al*., 2025).

Regardless of seasonality, Veredas are a net source of CH_4_. However, in a pristine Cerrado landscape, CH_4_ uptake by dry ecosystems can offset these emissions, as savannas can absorb 0.2 to 4.24 gC m^−2^ yr^−1^ (Cardoso *et al*., 2001; Siqueira Neto *et al*., 2011). Given that wetlands occupy *c*. 8% of the Cerrado, Vereda CH_4_ does not dominate regional budgets, even with higher wet‐season emissions. Yet, land‐use conversion reduces CH_4_ uptake (Cardoso *et al*., 2001) and can turn landscapes into net sources (Siqueira Neto *et al*., 2011). Since > 65% of areas surrounding Veredas are already impacted (Gonçalves *et al*., 2022), future land‐use change may shift regional CH_4_ balances toward net emissions.

Overall, the strong controls of precipitation, water‐table depth, and soil temperature on GHG fluxes demonstrate that Vereda carbon dynamics are highly sensitive to hydrological variation. Although carbon has accumulated over millennia, low carbon content in upper layers raises questions about whether recent changes in the hydrological cycle (Hofmann *et al*., 2021, 2023, 2025) are reducing accumulation rates or whether mineral inputs from disturbed landscapes are diluting surface carbon, potentially increasing vulnerability to peat burning.

### Conclusion: Vereda stores under threat

The long‐term carbon storage accumulated in Veredas and campos úmidos over the past 10 to 30 000 yr is increasingly threatened by anthropogenic land‐use changes, particularly drainage and conversion of native vegetation, which alter groundwater recharge and local hydrological connectivity (Durigan *et al*., 2022; Horák‐Terra *et al*., 2022b; Santos *et al*., 2023). There is growing pressure from agribusiness in the Cerrado, which not only converts grassland and savanna vegetation that plays a key role in groundwater recharge (Honda & Durigan, 2016; Oliveira *et al*., 2017) but also promotes large‐scale drainage of open wet grasslands and increases groundwater extraction through widespread, high‐water‐demand irrigation systems (e.g. center‐pivot irrigation; Althoff & Rodrigues, 2019; Latrubesse *et al*., 2019). These systems are strategically located around Veredas and campos úmidos due to the high water availability in their surroundings. As a result, even though Veredas and campos úmidos are legally protected, land‐use changes in the landscape are likely reducing water availability within these wetlands, with direct consequences for their carbon balance and long‐term stability.

These pressures are further intensified by climate change, which alters precipitation regimes and prolongs dry periods, exposing carbon to aerobic decomposition and accelerating GHG emissions (Santos *et al*., 2025). Our data revealed elevated CO_2_ emissions even during December, a typically wet month; this pattern is consistent with the delayed onset of rains during the 2023 El Niño event (CPTEC, 2023), highlighting how intensified climate events are already affecting Cerrado wetlands. A long‐term time series is necessary to corroborate this influence. Veredas are also significant sources of CH_4_, especially in permanently flooded areas, where emissions are amplified by rising temperatures. These ecosystems store carbon that is not only very old, but also chemically less stable than peat in wetter tropical regions, making them particularly vulnerable to even modest hydrological shifts. In addition, high‐intensity fires pose a major, yet largely overlooked, threat to Vereda peat, as extreme burns can ignite and consume peat, releasing long‐stored carbon; despite this risk, the vulnerability of Cerrado peat to deep burning remains almost largely uninvestigated (Turetsky *et al*., 2015).

When placing Veredas in a broader climatic context, we observe that they occur within the most extreme seasonality percentiles of tropical peatlands and receive substantially lower mean annual precipitation than most tropical ecosystems capable of accumulating peat. Veredas are also among the few groundwater‐fed, peat‐forming wetlands in the tropics, making them strongly dependent on local recharge processes rather than fluvial flooding or lacustrine inputs. This combination of high climatic seasonality and groundwater dependence suggests that Veredas operate near the threshold for peat formation and are therefore particularly vulnerable to shifts in local hydrology. The concurrent pressures of reduced groundwater recharge, large‐scale drainage, and intensifying climate extremes threaten to unlock an old and previously stable carbon pool, increasing net GHG emissions from the Cerrado.

Globally, the protection of carbon‐rich ecosystems has become a central pillar of climate policy (Keith *et al*., 2021). Yet, current strategies tend to prioritize forests, often overlooking non‐forest systems such as tropical savannas and open wetlands (Parr *et al*., 2014; Bond, 2016; Strassburg *et al*., 2017). In Brazil, while international attention focuses on the Amazon, agribusiness‐driven expansion continues to target the Cerrado, placing its biodiversity, water resources, and carbon stocks at serious risk (da Conceição Bispo *et al*., 2024). As the most biodiverse savanna on earth and the headwater region for two‐thirds of Brazil's major river basins, including rivers in the Amazon Basin, the Cerrado plays a critical role in both regional hydrology and global climate regulation (Wantzen *et al*., 2012; Strassburg *et al*., 2017; Soares *et al*., 2021; Rodrigues *et al*., 2022). Our findings underscore Veredas as one of Brazil's most carbon‐dense ecosystems, yet also among its most vulnerable to hydrological disturbance. Recognizing and protecting these peat‐forming wetlands is imperative, not only to strengthen national carbon accounting and meet global climate targets via nature‐based solutions, but also to secure water availability, safeguard biodiversity, and ensure the resilience of Brazil's most threatened biome.

## Competing interests

None declared.

## Authors contributions

LSV, DL‐M, NP, AEZ, and RSO planned and designed the research. LSV, GMA, and TA conducted the fieldwork. LSV and DH‐R conducted the laboratory analyses. LSV, PNB, JCFC, and GGM collected remote sensing data. LSV, PNB, DL‐M, and GGM performed the data analysis, and ST contributed to data analysis and interpretation. RSO supervised the project. LSV wrote the first version of the manuscript with significant support from RSO, ST, and AEZ. All authors contributed to revision and editing.

## Disclaimer

The New Phytologist Foundation remains neutral with regard to jurisdictional claims in maps and in any institutional affiliations.

## Supporting information


**Fig. S1** Monthly precipitation between 2010 and 2023 for Chapada dos Veadeiros region.
**Fig. S2** Description of sampling design.
**Fig. S3** Fourier‐transformed infrared spectra for Veredas sites.
**Fig. S4** Total carbon stocks across the soil profile for each studied Vereda.
**Fig. S5** Total carbon stocks across the soil profile for each studied Vereda.
**Fig. S6** Comparison of emissions inside and outside flooded areas (or inside and outside the Vereda) along the temporal series, spanning from January 2023 to February 2024.
**Fig. S7** Influence of ‘month’, ‘position’, and their interaction on CO_2_ and CH_4_.
**Fig. S8** Carbon emissions on Veredas along the sampled periods spanning from January 2023 to February 2024.
**Fig. S9** Influence of ‘month’, ‘flooding pattern’, and their interaction on CO_2_ and CH_4_.
**Methods S1** Extended methods on Veredas mapping.
**Methods S2** Extended methods on CO_2_ and CH_4_ efflux measurements.
**Table S1** Summary of soil samplings.
**Table S2** Test of model responses to different precipitation lag periods.
**Table S3** Random forest models' best parameters and weighted overall accuracy.
**Table S4** Total number of sampled units for each class, training points in each class for 10 models, and gross predicted area per class.
**Table S5** Parameters used to calculate the total carbon stock in each Vereda for samples with carbon content above 8%.
**Table S6** Accuracy estimates per class for 10 models used for cross‐validation.
**Table S7** ANOVA table for mixed‐effect models explaining the influence of position (inside or outside flooded area) and months on CO_2_ and CH_4_ fluxes.
**Table S8** Mixed‐effect models statistics results for models explaining CO_2_ and CH_4_ variation.
Please note: Wiley is not responsible for the content or functionality of any Supporting Information supplied by the authors. Any queries (other than missing material) should be directed to the *New Phytologist* Central Office.

## Data Availability

The data that support the findings of this study are openly available in Zenodo at doi: 10.5281/zenodo.17834473.
