# Supplementary material for: Vast, overlooked peat, and organic soils in Brazil's Cerrado: carbon storage, dynamics, and stability
Source: New Phytol. 2026 Mar 12;250(5):2946–65. doi: 10.1111/nph.71027 (PMC13150300; doi:10.1111/nph.71027)
Supplement: Supplementary file 1 — Fig. S1 Monthly precipitation between 2010 and 2023 for Chapada dos Veadeiros region. Fig. S2 Description of sampling design. Fig. S3 Fourier‐transformed infrared spectra for Veredas sites. Fig. S4 Total carbon stocks across the soil profile for each studied Vereda. Fig. S5 Total carbon stocks across the soil profile for each studied Vereda. Fig. S6 Comparison of emissions inside and outside flooded areas (or inside and outside the Vereda) along the temporal series, spanning from January 2023 to February 2024. Fig. S7 Influence of ‘month’, ‘position’, and their interaction on CO2 and CH4. Fig. S8 Carbon emissions on Veredas along the sampled periods spanning from January 2023 to February 2024. Fig. S9 Influence of ‘month’, ‘flooding pattern’, and their interaction on CO2 and CH4. Methods S1 Extended methods on Veredas mapping. Methods S2 Extended methods on CO2 and CH4 efflux measurements. Table S1 Summary of soil samplings. Table S2 Test of model responses to different precipitation lag periods. Table S3 Random forest models' best parameters and weighted overall accuracy. Table S4 Total number of sampled units for each class, training points in each class for 10 models, and gross predicted area per class. Table S5 Parameters used to calculate the total carbon stock in each Vereda for samples with carbon content above 8%. Table S6 Accuracy estimates per class for 10 models used for cross‐validation. Table S7 ANOVA table for mixed‐effect models explaining the influence of position (inside or outside flooded area) and months on CO2 and CH4 fluxes. Table S8 Mixed‐effect models statistics results for models explaining CO2 and CH4 variation. Please note: Wiley is not responsible for the content or functionality of any Supporting Information supplied by the authors. Any queries (other than missing material) should be directed to the New Phytologist Central Office. [file NPH-250-2946-s001.docx]

## *New Phytologist* Supporting Information

Article title: Vast, overlooked peat and organic soils in Brazil's Cerrado: carbon storage, dynamics, and stability

Authors: Larissa S Verona, Amy E Zanne, Susan Trumbore, Paulo Nx Bernardino, Guilherme M Alencar, Thalia Andreuccetti, David Herrera-Ramirez, João C F Cardoso, Demetrius Lira-Martins, Guilherme G Mazzochini, Natashi Pilon, Rafael S Oliveira

Article acceptance date: 15 January 2026.

The following Supporting Information is available for this article:

**Tables**

**Table S1.** Summary of soil sampling, including the number of samples collected per point, maximum sampling depth, indication of whether radiocarbon and FTIR analyses were performed, and geographic coordinates.

| **Site** | **Point** | **Samples** | **Max Depth (cm)** | **Radiocarbon** | **FTIR** | **Latitude** | **Longitude** |
| --- | --- | --- | --- | --- | --- | --- | --- |
| BBT | 1 | 3 | 200 | no | no | -14.09926 | -47.64708 |
| BBT | 2 | 5 | 250 | no | no | -14.09910 | -47.64757 |
| BBT | 3 | 4 | 170 | no | yes | -14.09877 | -47.64789 |
| BBT | 4 | 4 | 200 | no | no | -14.09862 | -47.64708 |
| BBT | 5 | 5 | 300 | yes | yes | -14.09844 | -47.64758 |
| BBT | 6 | 4 | 200 | no | yes | -14.09789 | -47.64815 |
| BBT | 7 | 5 | 250 | no | yes | -14.09799 | -47.64723 |
| BBT | 8 | 4 | 200 | no | no | -14.09790 | -47.64746 |
| BBT | 9 | 3 | 150 | no | no | -14.09774 | -47.64816 |
| ENG | 1 | 2 | 100 | no | no | -13.62188 | -47.47692 |
| ENG | 2 | 5 | 250 | no | yes | -13.62241 | -47.47680 |
| ENG | 3 | 2 | 65 | no | no | -13.62267 | -47.47696 |
| ENG | 4 | 2 | 100 | no | no | -13.62265 | -47.47585 |
| ENG | 5 | 4 | 200 | no | yes | -13.62312 | -47.47603 |
| ENG | 6 | 6 | 400 | yes | yes | -13.62318 | -47.47598 |
| ENG | 7 | 2 | 100 | no | yes | -13.62294 | -47.47440 |
| ENG | 8 | 4 | 220 | no | no | -13.62311 | -47.47418 |
| ENG | 9 | 1 | 50 | no | no | -13.62318 | -47.47404 |
| FDS | 1 | 8 | 350 | no | no | -14.19357 | -47.57780 |
| FDS | 2 | 7 | 315 | no | yes | -14.19352 | -47.57798 |
| FDS | 3 | 3 | 142 | no | yes | -14.19347 | -47.57815 |
| FDS | 4 | 4 | 160 | no | no | -14.19060 | -47.57796 |
| FDS | 5 | 8 | 320 | yes | yes | -14.19064 | -47.57816 |
| FDS | 6 | 4 | 325 | no | no | -14.19070 | -47.57835 |
| FDS | 7 | 3 | 150 | no | yes | -14.18674 | -47.57816 |
| FDS | 8 | 8 | 241 | no | yes | -14.18671 | -47.57833 |
| FDS | 9 | 3 | 100 | no | no | -14.18664 | -47.57859 |
| ING | 1 | 5 | 230 | no | yes | -14.14205 | -47.75203 |
| ING | 2 | 4 | 180 | no | yes | -14.14167 | -47.75103 |
| ING | 3 | 8 | 400 | yes | yes | -14.14112 | -47.75003 |
| ING | 4 | 6 | 250 | no | yes | -14.14128 | -47.74988 |
| ING | 5 | 3 | 150 | no | no | -14.14060 | -47.75042 |
| ING | 6 | 4 | 163 | no | yes | -14.14112 | -47.75146 |
| ING | 7 | 4 | 153 | no | no | -14.14037 | -47.75569 |
| ING | 8 | 5 | 280 | no | yes | -14.14054 | -47.75616 |
| ING | 9 | 7 | 400 | no | yes | -14.14092 | -47.75640 |
| ING | 10 | 3 | 200 | no | yes | -14.14264 | -47.75228 |
| RAJ | 1 | 5 | 186 | no | no | -14.14380 | -47.70122 |
| RAJ | 2 | 5 | 140 | no | yes | -14.14418 | -47.70118 |
| RAJ | 3 | 4 | 100 | no | yes | -14.14482 | -47.70121 |
| RAJ | 4 | 5 | 150 | no | no | -14.14516 | -47.70118 |
| RAJ | 5 | 5 | 75 | no | yes | -14.14242 | -47.70670 |
| RAJ | 6 | 6 | 400 | yes | yes | -14.14278 | -47.70689 |
| RAJ | 7 | 9 | 278 | no | yes | -14.14313 | -47.70708 |
| RAJ | 8 | 2 | 90 | no | yes | -14.14146 | -47.70898 |
| RAJ | 9 | 2 | 127 | no | no | -14.13436 | -47.71622 |
| RAJ | 10 | 5 | 250 | no | yes | -14.13445 | -47.71644 |
| RAJ | 11 | 4 | 200 | no | yes | -14.13448 | -47.71650 |
| VVT | 1 | 3 | 100 | no | yes | -14.18113 | -47.57131 |
| VVT | 2 | 5 | 150 | yes | yes | -14.18144 | -47.57140 |
| VVT | 3 | 4 | 100 | no | yes | -14.18148 | -47.57151 |
| VVT | 4 | 2 | 100 | no | no | -14.18164 | -47.5715 |
| VVT | 5 | 3 | 74 | no | yes | -14.18165 | -47.57144 |
| VVT | 6 | 2 | 60 | no | no | -14.18159 | -47.57122 |

**Table S2.** Test of model responses to different precipitation lag periods.

| **Gas** | **Lag** | **Marginal *r^2^*** | **Conditional *r^2^*** | ***p* value** | | | | | **logLik** | **AIC** |
| --- | --- | --- | --- | --- | --- | --- | --- | --- | --- | --- |
|  |  |  |  | *Precipitation* | *Soil Temperature* | *Flooding Pattern* | *Precipitation: Flooding Pattern* | *Soil Temperature: Flooding Pattern* |  |  |
| CO_2_ | 0 | 0.12 | 0.56 | 0.45 | <0.001 | 0.60 | 0.08 | 0.26 | -235 | 490 |
|  | 1 | 0.13 | 0.57 | 0.09 | 0.00 | 0.82 | 0.10 | 0.45 | -230 | 480 |
|  | 2 | 0.16 | 0.60 | 0.00 | 0.02 | 0.67 | 0.02 | 0.90 | **-212** | **444** |
|  | 3 | 0.14 | 0.58 | 0.05 | 0.01 | 0.99 | 0.06 | 0.57 | -224 | 468 |
|  | 4 | 0.11 | 0.55 | 0.54 | <0.001 | 0.38 | 0.59 | 0.19 | -239 | 499 |
|  | 5 | 0.11 | 0.55 | 0.61 | <0.001 | 0.20 | 0.35 | 0.12 | -239 | 498 |
|  | 6 | 0.12 | 0.56 | 0.34 | <0.001 | 0.34 | 0.08 | 0.24 | -234 | 488 |
| CH_4_ | 0 | 0.49 | 0.67 | 0.80 | <0.001 | 0.38 | 0.01 | <0.001 | **-265** | **548** |
|  | 1 | 0.48 | 0.67 | 0.91 | <0.001 | 0.79 | 0.01 | <0.001 | -266 | 549 |
|  | 2 | 0.47 | 0.66 | 0.34 | 0.01 | 0.88 | 0.03 | <0.001 | -271 | 559 |
|  | 3 | 0.47 | 0.66 | 0.62 | 0.00 | 0.20 | 0.71 | <0.001 | -273 | 565 |
|  | 4 | 0.48 | 0.66 | 0.83 | 0.00 | 0.04 | 0.24 | <0.001 | -272 | 562 |
|  | 5 | 0.48 | 0.66 | 0.99 | <0.001 | 0.03 | 0.04 | <0.001 | -269 | 556 |
|  | 6 | 0.49 | 0.67 | 0.91 | <0.001 | 0.06 | 0.01 | <0.001 | -266 | 551 |

Note: For each model, we used the sum of precipitation over three consecutive months. The last month included in the sum was determined by the difference between the lag month value (second column) and the actual collection date. We present the p-values, as well as the marginal and conditional R² for each model. Model selection was based on the highest log-likelihood and the lowest Akaike Information Criterion (AIC) values (best values are highlighted). We selected the lag based on the CO₂ best value, as there were no substantial differences between the best lag for CH₄ and the selected lag CH₄ model results.

**Table S3.** Random forest models' best parameters and weighted overall accuracy for each cross-validation model

| Model | Number of Variables | Number of Trees | Minimum Samples per Leaf | Overall Accuracy |
| --- | --- | --- | --- | --- |
| 1 | 15 | 800 | 1 | 83% (1.2) |
| 2 | 9 | 800 | 1 | 81% (0.6) |
| 3 | 15 | 1100 | 1 | 82% (0.6) |
| 4 | 9 | 500 | 1 | 81% (0.6) |
| 5 | 15 | 800 | 1 | 82% (0.6) |
| 6 | 15 | 800 | 1 | 80% (0.7) |
| 7 | 15 | 800 | 1 | 82% (0.7) |
| 8 | 9 | 800 | 1 | 83% (0.6) |
| 9 | 15 | 1100 | 1 | 83% (0.6) |
| 10 | 15 | 1100 | 1 | 84% (0.6) |

Note: Accuracy values and standard errors, in parentheses, were measured using a weighted confusion matrix.

**Table S4.** Total number of sampled units for each class, training points in each class for 10 models, and gross predicted area per class.

| Class | Sampled Points | Testing Points | | | | | | | | | | Predicted Area (Mha) |
| --- | --- | --- | --- | --- | --- | --- | --- | --- | --- | --- | --- | --- |
|  |  | Model 1 | Model 2 | Model 3 | Model 4 | Model 5 | Model 6 | Model 7 | Model 8 | Model 9 | Model 10 |  |
| Vereda | 3186 | 738 | 651 | 734 | 851 | 666 | 922 | 872 | 576 | 816 | 991 | 19,33 |
| Agriculture | 4279 | 1101 | 1136 | 977 | 1039 | 1075 | 1008 | 1082 | 982 | 983 | 1147 | 64,38 |
| Water | 616 | 190 | 172 | 178 | 131 | 165 | 133 | 141 | 132 | 136 | 95 | 1,51 |
| Grassland | 1684 | 423 | 410 | 441 | 384 | 413 | 436 | 381 | 392 | 384 | 330 | 14,72 |
| Savanna | 2541 | 805 | 661 | 743 | 592 | 556 | 502 | 504 | 689 | 819 | 460 | 38,46 |
| Riparian Forest | 1691 | 352 | 395 | 337 | 393 | 555 | 441 | 382 | 483 | 175 | 306 | 11,21 |
| Forest | 525 | 80 | 190 | 80 | 256 | 127 | 124 | 147 | 204 | 402 | 161 | 1,26 |
| Constructed Area | 93 | 9 | 8 | 10 | 7 | 67 | 68 | 9 | 10 | 17 | 15 | 0,74 |
| Forestry | 338 | 40 | 115 | 106 | 91 | 57 | 53 | 66 | 97 | 78 | 38 | 2,04 |
| Total | 14953 | 3738 | 3738 | 3606 | 3744 | 3681 | 3687 | 3584 | 3565 | 3810 | 3543 | 153,66 |

**Table S5.** Mean (± 95% Confidence Interval) of parameters used to calculate the total carbon stock in each Vereda, considering only samples with carbon content above 8% (the threshold for organic soil in Brazilian soil classification).

| **Site** | **Mean Peat Thickness (m)** | **Mean C (%)** | **Dry Bulk Density (kg/m³)** | **Area (m²)** | **C Density (Mg/ha)** | **Max C Density (Mg/ha)** | **Min C Density (Mg/ha)** |
| --- | --- | --- | --- | --- | --- | --- | --- |
| BBT | 1.63 (± 0.26) | 13.4 (± 1.2) | 593.78  (± 122.32) | 3.98 | 932 (±280) | 1572.29 | 177.36 |
| ENG | 0.78 (± 0.29) | 17.6 (± 7.1) | 342.23 (± 86.19) | 3.38 | 509 (±300) | 712.94 | 208.84 |
| FDS | 1.72 (± 0.86) | 13.7 (± 2.2) | 749.55  (± 174.75) | 4.06 | 1,341 (±713) | 2,481.00 | 145.16 |
| ING | 2.22 (± 0.65) | 15.5 (± 2.7) | 563.36  (± 149.42) | 16.94 | 1,114 (±404) | 2,367.89 | 251.65 |
| RAJ | 1.48 (± 0.55) | 19 (± 2.3) | 752.91  (± 201.90) | 28.69 | 1,370 (±598) | 3,545.49 | 144.27 |
| VVT | 0.62 (± 0.34) | 20.2 (± 5.9) | 521.04  (± 269.24) | 0.39 | 595 (±290) | 1,681.93 | 47.95 |

Note: Confidence intervals were calculated for Mean Thickness and Carbon Density at the point level, and for Mean C and Dry Bulk Density at the sample level. Data for area, Max and Min C density are absolute values. Data for Area, Max, and Min C density are absolute values.

**Table S6.** Accuracy estimates per class for 10 models used for cross-validation.

|  | Vereda | Agriculture | Water | Grassland | Savanna | Riparian Forest | Forest | Constructed Area | Forestry |
| --- | --- | --- | --- | --- | --- | --- | --- | --- | --- |
| **Model 1** |  |  |  |  |  |  |  |  |  |
| Precision | 90% (1.2) | 95% (0.5) | 73% (5.1) | 62% (2) | 82% (1.1) | 78% (2.3) | 21% (3.2) | 65% (13) | 75% (5.3) |
| Recall | 75% (1.6) | 93% (0.8) | 98% (1.1) | 75% (2.3) | 72% (1.5) | 67% (2.2) | 45% (7) | 56% (18) | 86% (6.5) |
| **Model 2** |  |  |  |  |  |  |  |  |  |
| Precision | 86% (1.4) | 92% (0.8) | 94% (1.7) | 79% (0.2) | 66% (1.6) | 64% (2.1) | 76% (6.9) | 20% (20) | 96% (0.2) |
| Recall | 82% (1.6) | 95% (0.6) | 67% (6.6) | 62% (1.9) | 86% (1.2) | 74% (0.2) | 15% (1.5) | 28% (21) | 53% (4) |
| **Model 3** |  |  |  |  |  |  |  |  |  |
| Precision | 83% (1.3) | 92% (0.8) | 93% (1.7) | 73% (2.1) | 74% (1.6) | 66% (2.3) | 29% (5.2) | NA | 83% (5.2) |
| Recall | 77% (1.5) | 97% (0.4) | 84% (4.6) | 63% (2.1) | 80% (1.1) | 71% (2.5) | 14% (2.7) | NA | 46% (3.8) |
| **Model 4** |  |  |  |  |  |  |  |  |  |
| Precision | 86% (1.1) | 95% (0.6) | 93% (2.2) | 71% (2.2) | 65% (1.9) | 56% (2) | 82% (8.4) | 75% (1.6) | 79% (5.6) |
| Recall | 81% (1.6) | 97% (0.4) | 83% (5.4) | 68% (2.3) | 82% (1.1) | 74% (3) | 10% (1.1) | 89% (9.2) | 55% (4.4) |
| **Model 5** |  |  |  |  |  |  |  |  |  |
| Precision | 77% (1.6) | 95% (0.6) | 95% (1.8) | 77% (2.1) | 66% (1.8) | 77% (1.8) | 44% (6.2) | 100 | 80% (5.2) |
| Recall | 78% (1.7) | 97% (0.4) | 70% (5.5) | 68% (2) | 87% (1) | 63% (2.2) | 12% (1.8) | 23% (2.6) | 79% (5.4) |
| **Model 6** |  |  |  |  |  |  |  |  |  |
| Precision | 85% (1.1) | 92% (0.8) | 94% (2.1) | 69% (2.5) | 61% (1.9) | 77% (1.9) | 52% (7.2) | 100 % | 90% (0.4) |
| Recall | 82% (1.6) | 96% (0.5) | 81% (4.9) | 51% (2) | 85% (1.3) | 75% (2.5) | 14% (2.1) | 21% (2.3) | 73% (5.6) |
| **Model 7** |  |  |  |  |  |  |  |  |  |
| Precision | 90% (1) | 92% (0.8) | 96% (1.6) | 81% (0.2) | 68% (0.2) | 68% (0.2) | 56% (0.6) | 57% (20) | 85% (4.5) |
| Recall | 82% (1.6) | 97% (0.5) | 74% (6.6) | 59% (2.1) | 86% (1.2) | 76% (2.7) | 14% (1.8) | 61% (14) | 71% (5.6) |
| **Model 8** |  |  |  |  |  |  |  |  |  |
| Precision | 86% (1.5) | 97% (0.6) | 96% (1.7) | 75% (2.4) | 66% (1.6) | 66% (1.9) | 53% (6.4) | 86% (14) | 80% (4) |
| Recall | 84% (1.5) | 96% (0.4) | 85% (5) | 62% (2) | 86% (1.1) | 67% (2.4) | 11% (1.4) | 72% (11) | 71% (6.5) |
| **Model 9** |  |  |  |  |  |  |  |  |  |
| Precision | 86% (1.5) | 97% (0.6) | 96% (1.7) | 75% (2.4) | 66% (1.6) | 66% (1.9) | 53% (6.4) | 86% (14) | 80% (4) |
| Recall | 84% (1.5) | 96% (0.4) | 86% (4.8) | 62% (2.5) | 86% (1) | 67% (2.4) | 11% (1.4) | 72% (11) | 71% (5.9) |
| **Model 10** |  |  |  |  |  |  |  |  |  |
| Precision | 95% (0.7) | 94% (0.6) | 88% (3.2) | 81% (2.3) | 71% (1.9) | 62% (2.4) | 65% (7.3) | 80% (13) | 71% (6.8) |
| Recall | 80% (1.6) | 96% (0.6) | 90% (5) | 69% (2.3) | 91% (0.9) | 72% (3.1) | 13% (1.6) | 60% (9.9) | 83% (6.1) |

Note: Accuracy values and standard errors, in parentheses, were measured using a weighted confusion matrix.

**Table S7.** Anova table for mixed-effect models explaining the influence of position (inside or outside flooded area) and months on CO_2_ and CH_4_ fluxes.

| **log(CO_2_ + 756) ~ position * month + (1 \| site / point / repetition)** | | | | | |
| --- | --- | --- | --- | --- | --- |
|  | *df* | *SS* | *MS* | *F Value* | *p* |
| *position* | 1 | 2.58 | 2.58 | 17.00 | **<0.001** |
| *month* | 5 | 12.46 | 2.49 | 16.40 | **<0.001** |
| *position : month* | 5 | 7.43 | 1.48 | 9.78 | **<0.001** |
| **log(CO_2_ + 756) ~ month * pattern + (1 \| site / point / repetition)** | | | | | |
|  | *df* | *SS* | *MS* | *F Value* | *p* |
| *month* | 5 | 20.13 | 04.02 | 23.04 | **<0.0001** |
| *pattern* | 1 | 0.16 | 0.16 | 0.92 | 0.35 |
| *month : pattern* | 5 | 1.69 | 0.33 | 1.93 | 0.08 |
| **log(CH_4_ + 2.3 ) ~ position * month + (1 \| site / point / repetition)** | | | | | |
|  | *df* | *SS* | *MS* | *F Value* | *p* |
| *position* | 1 | 57.61 | 57.61 | 290.95 | **<0.001** |
| *month* | 5 | 6.17 | 1.23 | 6.23 | **<0.001** |
| *position : month* | 5 | 1.83 | 0.36 | 1.85 | 0.10 |
| **log(CH_4_ + 2.3 ) ~ month * pattern + (1 \| site / point / repetition)** | | | | | |
|  | *df* | *SS* | *MS* | *F Value* | *p* |
| *month* | 5 | 2.34 | 0.46 | 2.82 | **0.01** |
| *pattern* | 1 | 5.47 | 5.47 | 33.09 | **<0.001** |
| *month : pattern* | 5 | 6.89 | 1.38 | 8.33 | **<0.001** |

Note: Df: degrees of freedom, SS: sum of squares, MS are mean square. P-values above 0.05 are highlighted in bold.

**Table S8.** Mixed-effect models statistics results for models explaining CO_2_ and CH_4_ variation.

| **log(CO_2_ + 756) ~ preciptation*flooding pattern + soil temperature + (1 \| site / point / repetition)** | | | |
| --- | --- | --- | --- |
|  | *Estimates* | *CI* | *p* |
| *Intercept* | 7.10 | 6.65 – 7.55 | **<0.001** |
| *preciptation* | -3.65 * 10^-4^ | -4,71 * 10^-4^ – -2,58 * 10^-4^ | **<0.001** |
| *soil temperature* | 0.02 | 0.01 – 0.03 | **<0.001** |
| *preciptation : flooding pattern (seasonally)* | -3.15 * 10^-4^ | -4.38 * 10^-4^ – -1.92 * 10^-4^ | **0.01** |
| **log(CH_4_ + 2.3) ~ preciptation*pattern + soil temperature*pattern + (1 \| site / point / repetition)** | | | |
|  | *Estimates* | *CI* | *p* |
| *Intercept* | 1.19 | 0.80 – 1.58 | **<0.001** |
| *preciptation : flooding pattern (permanently)* | -1.36 * 10^-4^ | -2.55 * 10^-4^ – -0,17 * 10^-4^ | 0.25 |
| *preciptation : flooding pattern (seasonally)* | 2.53 * 10^-4^ | 1.53 * 10^-4^ – 3.53 * 10^-4^ | **0.01** |
| *soil temperature : flooding pattern (permanently)* | 0.03 | 0.02 – 0.04 | **<0.001** |
| *soil temperature : flooding pattern (seasonally)* | -0.01 | -0.03 – 0.00 | **0.05** |

Note: The initial models including precipitation*pattern + soil temperature*pattern were simplified through backward selection. CI: 95% Confidence Intervals. P-values equal or below 0.05 are highlighted in bold.

**Figures**

**
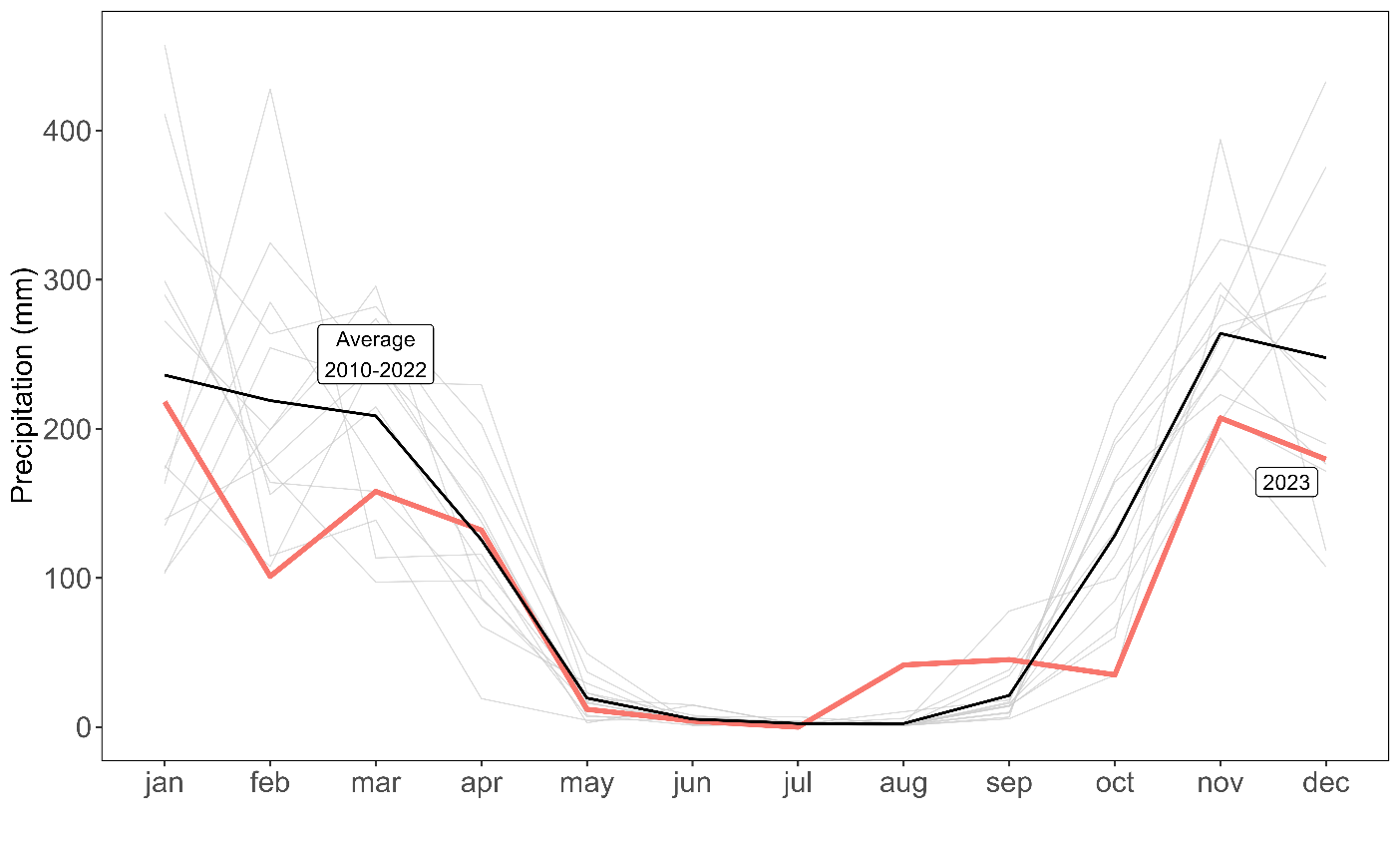
**

**Fig S1.** Monthly precipitation between 2010-2023 for Chapada dos Veadeiros region. The average precipitation between 2010 and 2023 is highlighted in black, while the precipitation of 2023 (when the samples were collected) is highlighted in red. The data were obtained from the WorldClim database (for 2010 – 2019) and local meteorological towers (2022 – 2023).

*
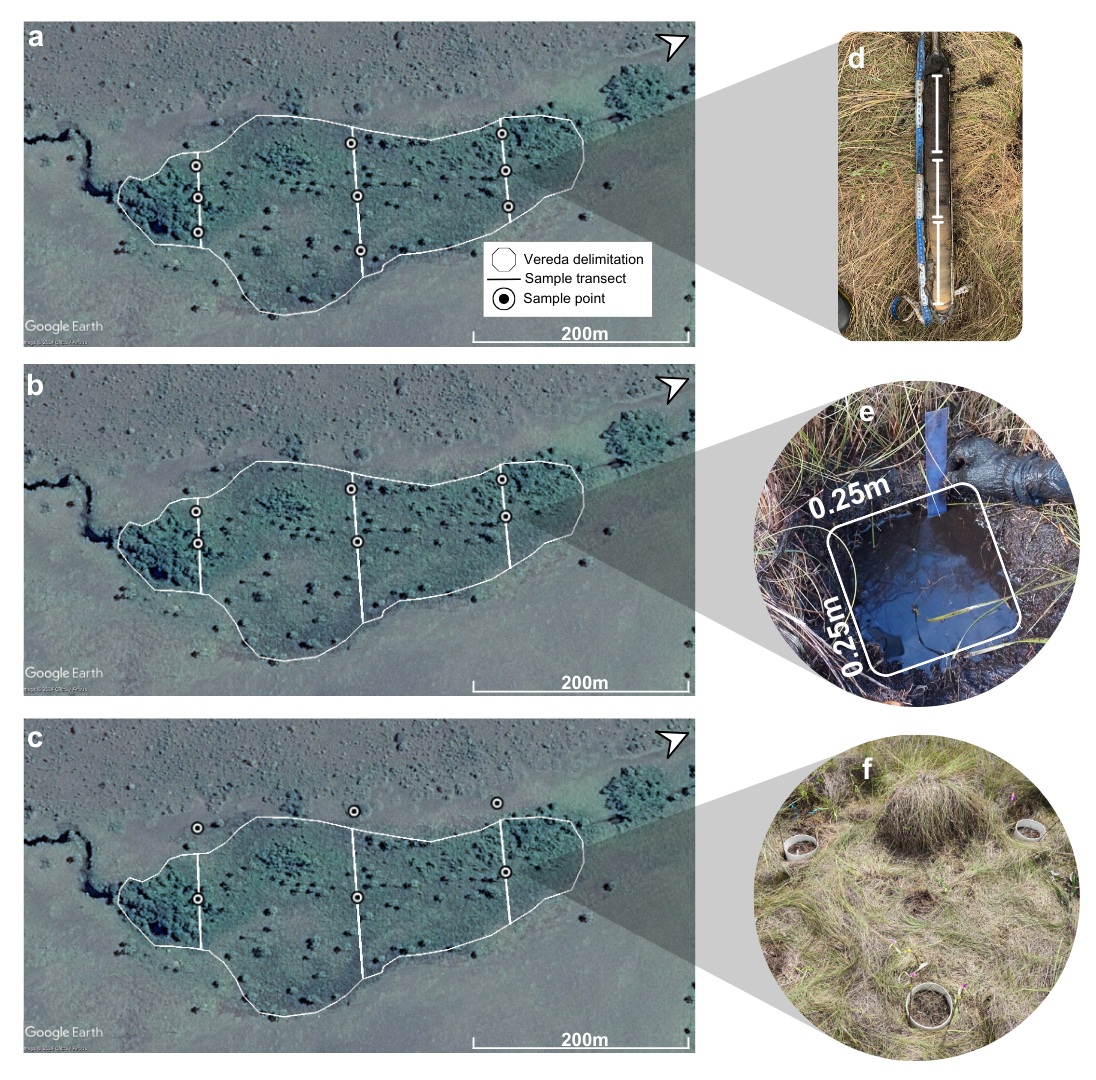
*

**Fig S2.** Description of sampling design. Panels *a)*, *b)*, and *c)* sampling points for soil, biomass, and flux, respectively. In these panels, the polygon represents the delimited Vereda outlined by RGB and microwave images; lines indicate each transect perpendicular to water flux; and circles represent the sampling points. Panel *d)* illustrates soil sampling using a Russian Peat Auger. The white bars highlight different layers, with one soil sample obtained per layer. Panel *e)* illustrates subplots for herbaceous biomass sampling, both above and below ground, with biomass collected up to 0.2 m in below-ground sampling. Panel *f)* demonstrates the three spatial replications at each point for soil flux sampling. CO_2_ and CH_4_ fluxes were measured twice in each ring over 90 seconds, and the accumulation curve slope was utilized to quantify the fluxes. For further details, please refer to the methods section.


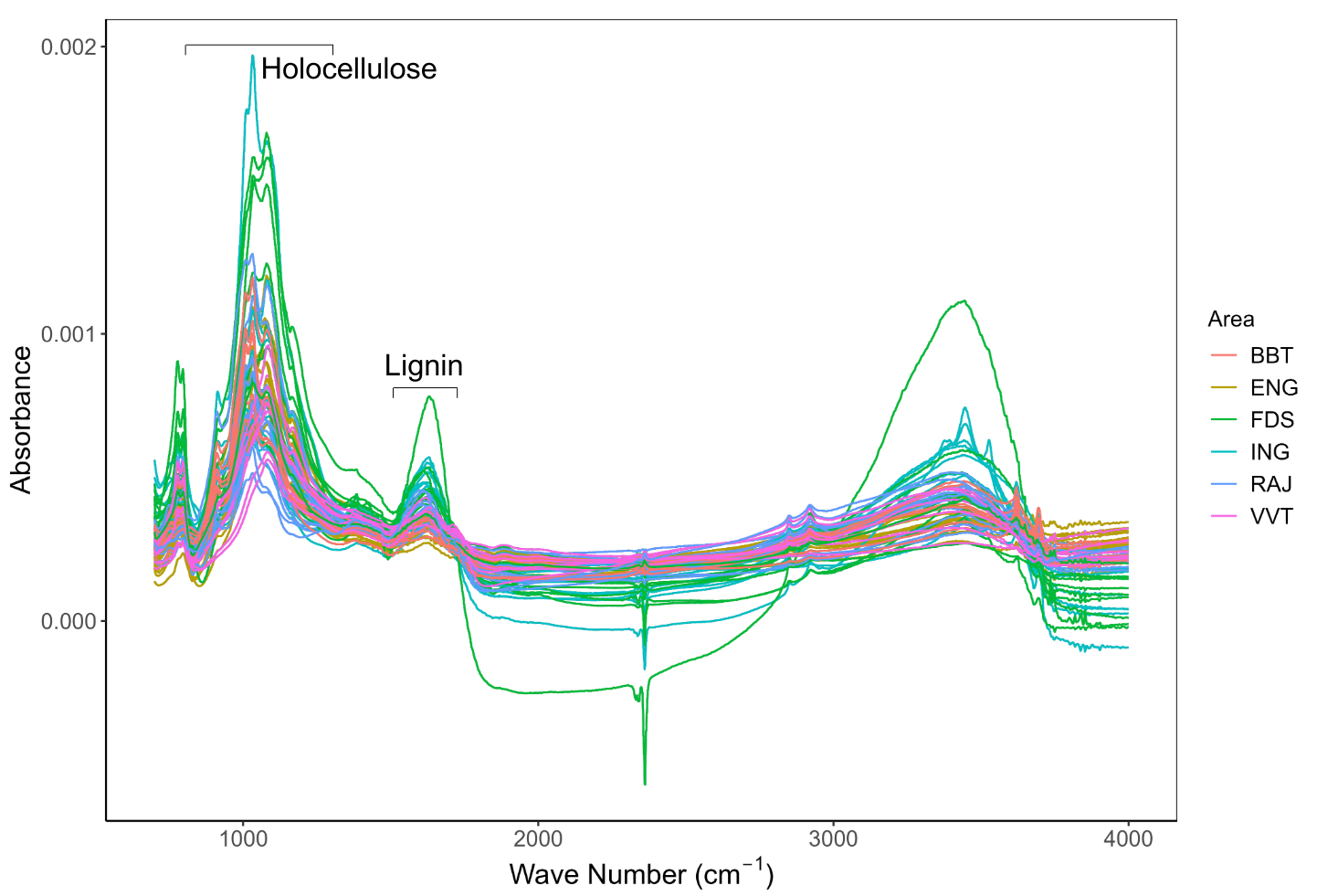


**Fig S3.** Fourier-transformed infrared spectra for 60 samples of organic soils from six Veredas. The Holocellulose and Lignin contents were obtained by the total area in peaks on wave numbers 1,030 nm and 1,510 nm – 1,630 nm, as highlighted in the plot after basal normalization.


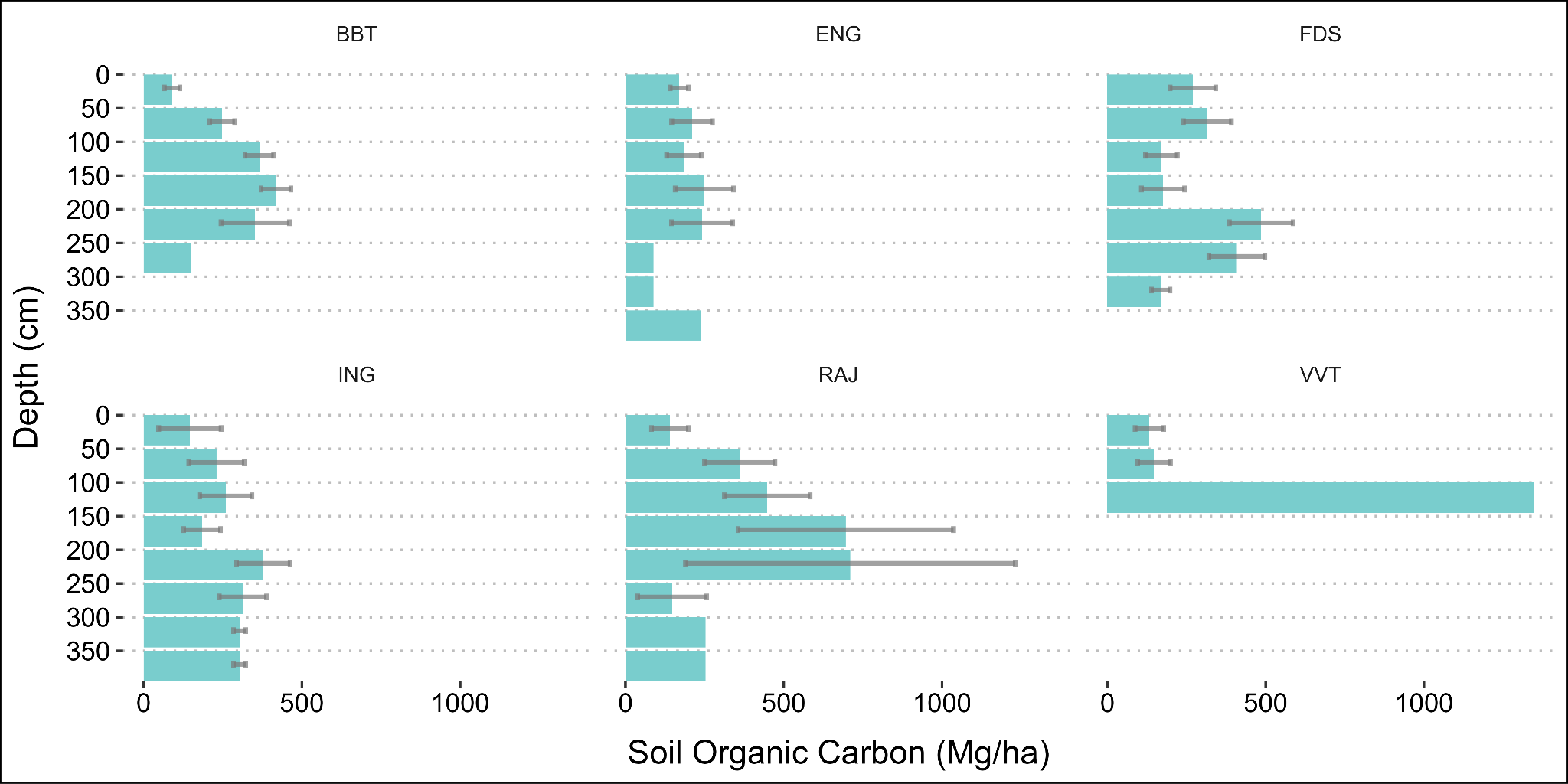


**Fig S4.** Total carbon stocks across the soil profile for each studied Vereda. Values represent means per point, and error bars indicate the standard error.


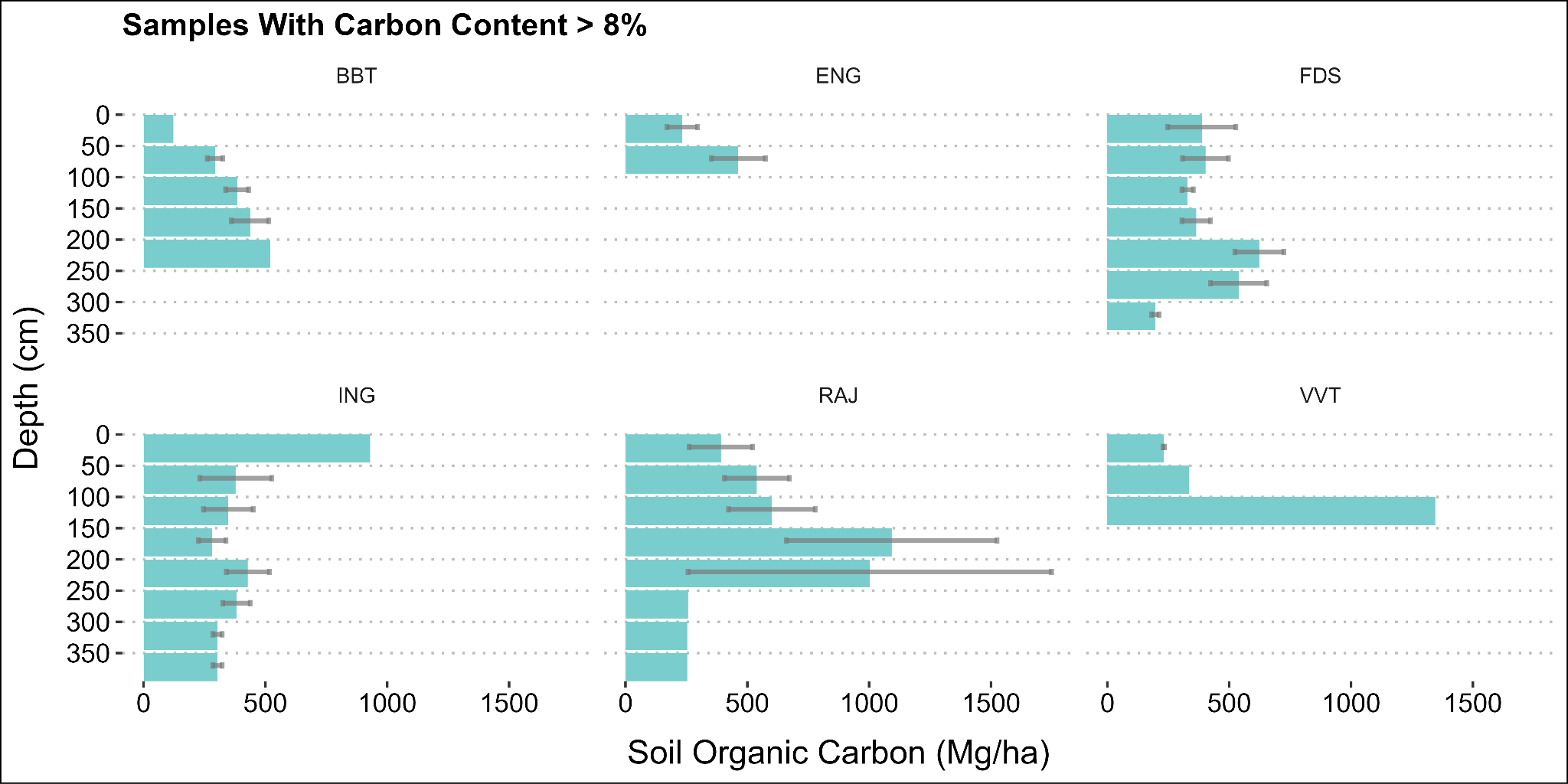


**Fig S5.** Total carbon stocks across the soil profile for each studied Vereda. We only added samples with carbon content above the threshold of 8% for organic soils in the Brazilian soil classification. Values represent means per point, and error bars indicate the standard error.


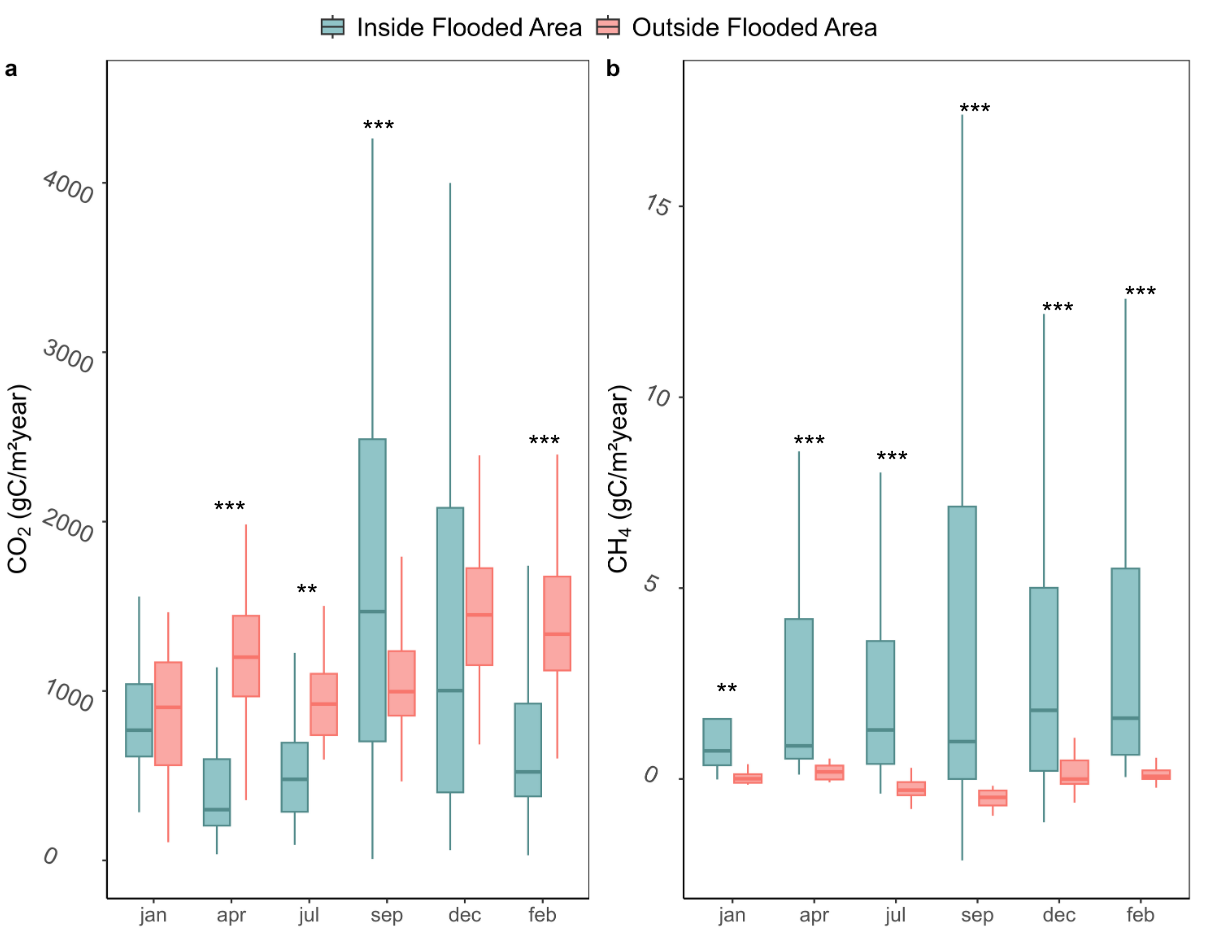


**Fig S6**. Comparison of emissions inside (green) and outside (red) flooded areas (or inside and outside the Vereda) along the temporal series, spanning from January 2023 to February 2024. Panel *a)* shows the CO_2_ emissions and panel *b)* shows CH_4_ emissions. Note the different units for CO_2_ and CH_4_. Statistical differences between groups in linear mixed-effect models are highlighted by asterisks with *** representing *p*<0.001 and ** representing *p* < 0.01.


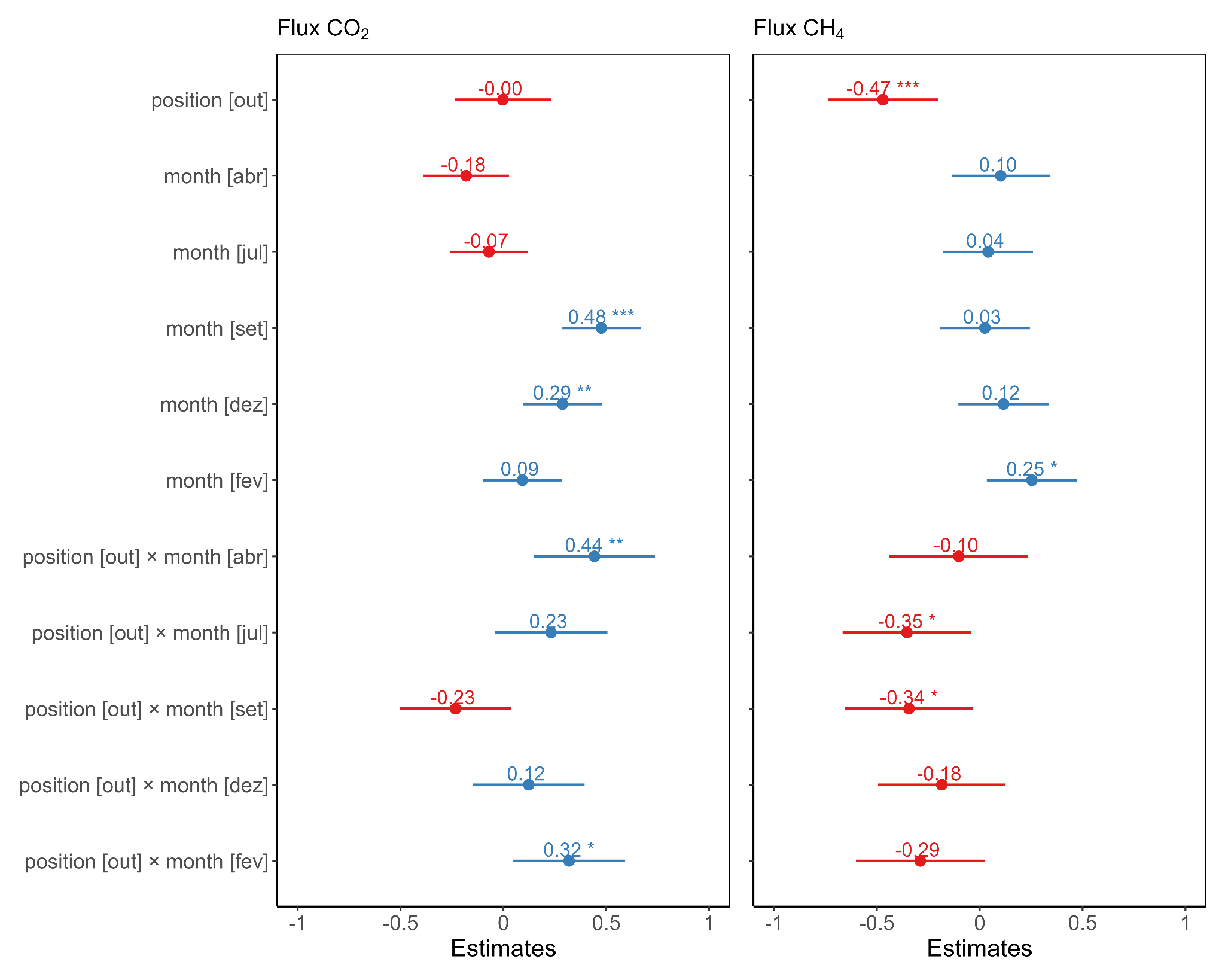


**Fig S7.** Influence of ‘month’, ‘position’, and their interaction on CO_2_ (left panel) and CH_4_ (right panel). The blue values denote positive estimates and the red values represent negative ones. Statistical significance in linear mixed-effect models is highlighted by asterisks with *** representing *p*<0.001, ** representing *p* < 0.01, and * representing p<0.05.


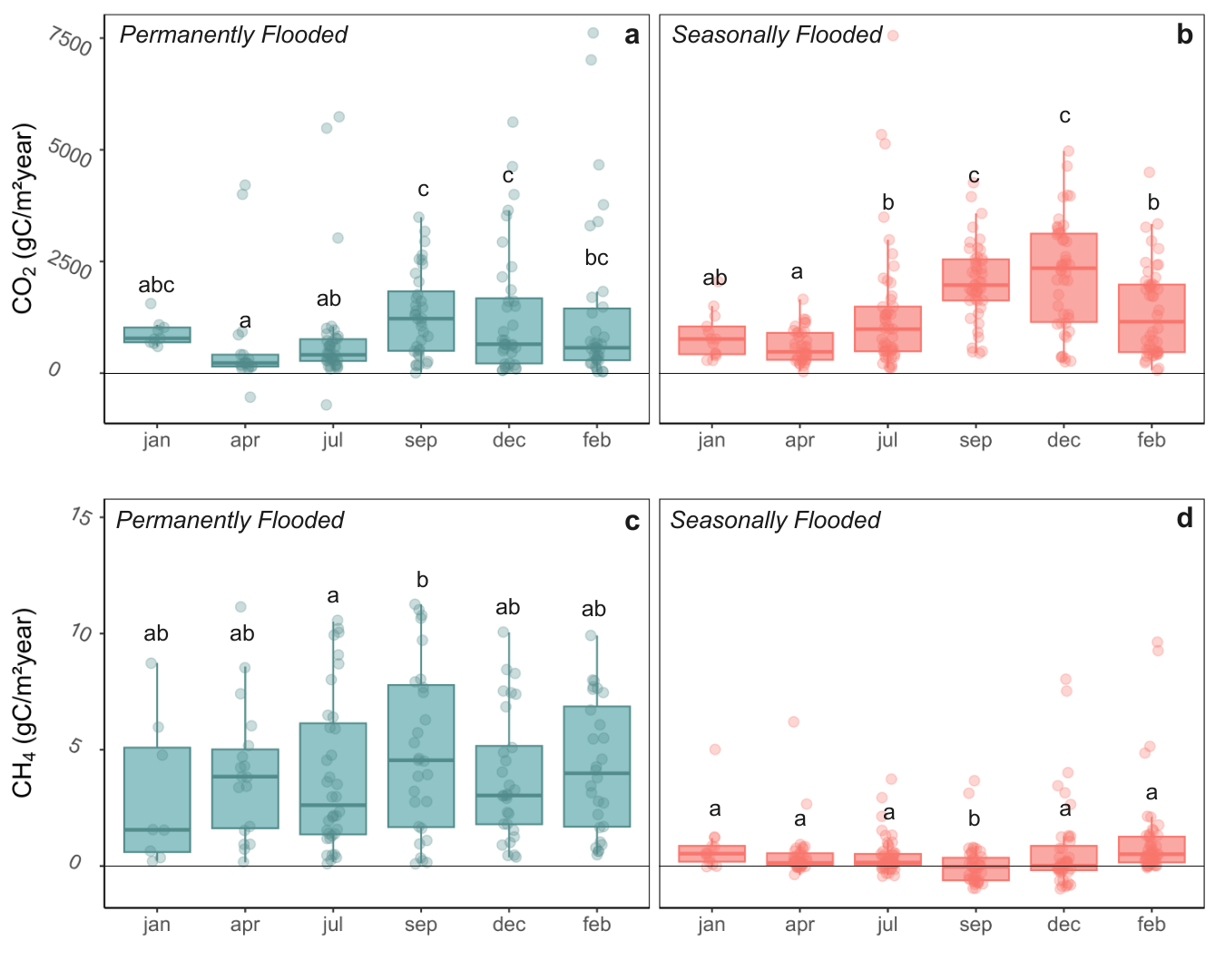


**Fig S8.** Carbon emissions on Veredas along the sampled periods spanning from January 2023 to February 2024. July, September, and December are the drier months. The letters denote statistical differences among months within each group, as determined by linear mixed-effect models with '*months * flooding pattern*' as predictors and post-hoc pairwise comparisons through Estimated Marginal Means. The boxes indicate the interquartile range (first to third quartiles), and the horizontal line within each box represents the median. Panels *a* and *b* represent CO_2_ emissions, while panels *c* and *d* represent CH_4_ emissions. Panels *a)* and *c)* display emissions from Veredas flooded year-round, while panels *b)* and *d)* show emissions from Veredas flooded only during wet months. Note the different units for CO_2_ and CH_4_ emissions.


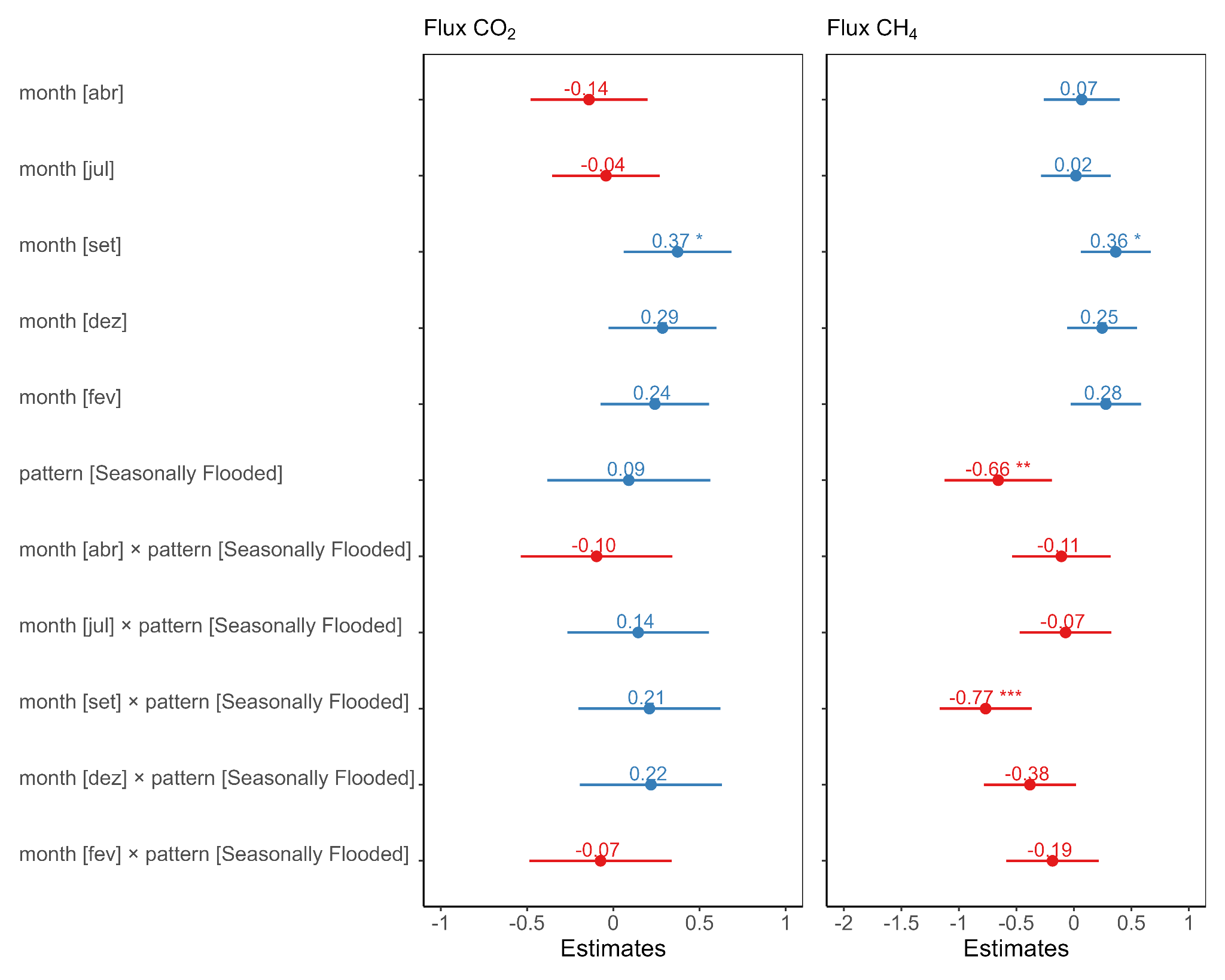


**Fig S9.** Influence of ‘month’, ‘flooding pattern’, and their interaction on CO_2_ (left panel) and CH_4_ (right panel). The blue values denote positive estimates and the red values represent negative ones. Statistical significance in linear mixed-effect models is highlighted by asterisks with *** representing *p*<0.001, ** representing *p* < 0.01, and * representing p<0.05.

**Methods 1. Extended methods on Veredas mapping**

To predict the spatial extent of Veredas across the Cerrado, we performed a land cover classification using a supervised Random Forest model. We obtained 15,008 reference points for land cover classes, of which 3,208 correspond to Veredas. Most Vereda points were obtained from the Brazilian Rural Environmental Register dataset (SICAR, 2023), which provides publicly available, self-reported boundaries of legally protected areas (including Veredas) within rural properties. Since landowners have no explicit incentive to map protected areas in detail, it is reasonable to expect that only areas clearly recognized as Veredas were registered, which increases confidence in the classification. Data download was manually conducted individually for each municipality between 26 July 2023 and 04 October 2023. We randomly subsampled the entire database of 97,877 polygons to 2,067. To reduce inconsistencies in the self-reported land-use classes in SICAR, we performed a second validation using a visual interpretation of true color images from Google Earth Pro, including Airbus, CNES Airbus and Landsat images (Google, 2025). For each subsampled and validated Vereda polygon, we sampled training points within the polygon boundaries. The land cover types surrounding Veredas were classified into 8 classes (‘agriculture’, ‘water’, ‘grassland’, ‘savanna’, ‘riparian forest’, ‘forest’, ‘constructed area’ and ‘forestry’) also through visual interpretation from Google Earth true color imagery (Google, 2025). We first identified the land cover types present in the landscape within an approximately 30-km radius, and then randomly selected points in each land cover type. This database was merged with a classification database for Chapada dos Veadeiros region obtained by Lewis et al. (2022). Only 25% of the Lewis database was used to balance the proportion of points in our dataset with the spatial representativeness of the region. We limited the classification area to locations with reference points, removing areas north or south of the last reference point. These data were used to perform a supervised classification using Random Forest algorithms (Gislason et al., 2006), adapted from Lewis et al., (2022).

We generated quarterly median composites of Sentinel-2 and Sentinel-1 imagery for a delimited area including the entire Cerrado domain (limits: -25.0°S -60.5°W, -25.0°S -41.3°W, -2.33°S -41.3°W, -2.33°S -60.5°W). Sentinel-1 radar data (VV and VH backscatter) were filtered for the Interferometric Wide Swath (IW) instrument mode and descending orbits. Sentinel-2 surface reflectance data (B2, B3, B4, B5, B6, B7, B8, B11, B12) were processed to mask clouds and cirrus using the 'QA60' band and filtered for <50% cloud cover. Data from 2018-2023 were grouped into four quarterly periods, merged, and aggregated to compute median composites for each quarter. We extracted pixel values from raster data by creating 15 m buffers around sampling points and linking these areas to vegetation types. We also calculated indices to be included as predictors in the training of the Random Forest algorithm: the Normalized Difference Vegetation Index (NDVI; Tucker, 1979), difference between NDVI in August (dry) and January (wet) season, mid-infrared band ratio (SWIR2/SWIR1; Misra et al., 2020), and polarization ratio (VH/VV; Dubois et al., 2020). We obtained Digital Elevation Models (DEM) from NASA SRTM Digital Elevation product and calculated the mean elevation difference of pixels distant 1 km, 500 m and 200 m of each pixel using a squared kernel. Finally, we calculated the Topographic Wetness Index (Beven and Kirkby, 1979; Riihimäki et al., 2021) for each pixel from the DEM using the withebox R package (Wu, 2022), totaling 62 predictors for training the Random Forest. Final composites were exported with 30 m resolution. Image processing and exporting were conducted in Google Earth Engine.

Spatial autocorrelation of the response variables was assessed using the *blockCV* package in R (Valavi et al., 2019). We found that training points closer than 87 km exhibited spatial autocorrelation; therefore, we subset our training data into blocks of 90 km² to avoid spatial dependence. Using this block structure, approximately 25% of the points were assigned to model validation. As the number of points within blocks was not homogeneous, we generated four random subsets of blocks and selected the partition that most closely approximated the 25%/75% validation-to-training proportion. To perform spatial cross-validation, this procedure was repeated ten times, each time selecting different groups of training and validation blocks while preserving spatial independence (Roberts et al., 2017). We then fitted ten Random Forest models, classifying points into the following land-cover categories: “Vereda”, “grassland”, “savanna”, “riparian forest”, “forest”, “agriculture”, “forestry”, and “constructed area”. The hyperparameters “number of trees”, “number of variables”, and “minimum samples per leaf” were tuned for each model, and an internal 5-fold cross-validation was applied (see Tables S3 and S4 for details on each model). The resulting ten models were used to compute performance metrics (their mean and standard deviation are presented in the Results). Finally, to generate the classification map, we trained a final model using all available points (Roberts et al., 2017), applying the mode of the best selected hyperparameters: 1,100 trees, 15 variables at each split, and one as the minimum number of samples required in a node before it could be split.

We obtained performance metrics using the good practices protocol of Olofsson et al. (2014). From the testing samples, we estimated the population metrics and variances for each model. To do so, we calculated the weighted prediction for each class according to the equation below, obtaining an estimated population confusion matrix (Olofsson et al., 2014):

$$\hat{p_{ij}}=W_{i}\frac{n_{ij}}{n_{i}.}$$

Where, $\hat{p_{ij}}$ is the estimated population values predicted for class *i* that is referenced in class *j, W_i_* is the proportion of final map predicted as class *i*, *n_ij_* is the number of pixels predicted as class i and referenced in class j, and *n_i._* is the total number of pixels predicted as class *i*. Then, we calculated the accuracy metrics across the ten model runs, as well as the variances according Olofsson et al. (2014). To account for differences in variance both within and among, we calculated the metrics’ variance according equation below (Burnham and Anderson, 2004):

$$\hat{var}\left( \bar{\theta} \right)=\left[ \sum_{i=1}^{10} {\frac{1}{10}[\hat{var}\left( \hat{\theta_{i}} \right)+(\hat{\theta_{i}}- {\hat{\bar{\theta}})}^{2}]}^{\frac{1}{2}} \right]^{2}$$

where the variance of each metric ($\hat{\theta_{i}}$) for the ten models was estimated based on the mean of the models (*i*) internal variance measured (first term in the equation) and the variance among models (second term in equation) (Olofsson et al., 2014). The final estimated variance was used to calculated confidence intervals for the metrics. The evaluated metrics were: “overall accuracy” and “class accuracy” ([true positives + true negatives] / total), class “precision” or “user’s accuracy” (true positives / [true positives + false positives]), class “recall” or “producer’s accuracy” (true positives / [true positives + false negatives]), and “F1 scores” ([2 * precision*recall] / [precision + recall]). We corrected the Veredas prediction using the Vereda “precision” value and obtained confidence intervals using the Vereda “precision” confidence intervals. Finally, to verify that Veredas in different climate zones were indeed accumulating peat, we overlapped 177 ground-truth points of peat occurrence - sourced from previous records (Beer et al., 2024) and our own field sampling - into the generated map. All analyses were performed using R software (R Core Team, 2025) and Google Earth Engine with the packages tidymodels (Kuhn and Wickham, 2020), sf (Pebesma, 2018) and terra (Hijmans, 2025).

To obtain an indication of the potential carbon stock across all areas classified as Vereda, assuming that carbon accumulation occurs at the same rate as in our sampling sites and in previous studies, we estimated the total carbon stock of Brazilian Veredas as a function of the mapped Vereda extent and mean carbon storage. We calculated the mean carbon storage using our measurements and previously published datasets for the first 30 cm of soil, and we also used our own measurements for the full soil profile. To account for variability within and among measurements, we applied the same variance equation described above for performance metrics. Additionally, to incorporate both the uncertainty associated with the land cover mapping and the uncertainty in carbon measurements, we used the equation below (Wu et al., 2006):

$var \left( C_{stock} \right)= \sigma_{area}^{2}*\sigma_{Cdensity}^{2}+{(\mu_{area})}^{2}* \sigma_{Cdensity}^{2}+ {(\mu_{Cdensity})}^{2}* \sigma_{area}^{2}$

where $\sigma_{area}^{2}$ and $\sigma_{Cdensity}^{2}$ are the variances of the predicted area and carbon density, respectively, and $\mu_{area}$ and $\mu_{Cdensity}$ are their corresponding means. We acknowledge the limitations of this extrapolation, as the actual proportion of Veredas that are actively accumulating carbon remains unknown.

**Methods 2. Extended methods on CO_2_ and CH_4_ efflux measurements**

The CO₂ and CH₄ efflux measurements were conducted at five Vereda sites over six sampling months (January, April, July, and December 2023, and February 2024). At each site, we measured three points located in the central portion of the waterlogged area, following a longitudinal transect parallel to the water flow. We also measured three paired points outside the waterlogged zone (except at the VVT site, where no non-waterlogged grassland was found near the central points) to assess the effect of flooding on gas emissions (Fig. S2c).

To deal with small spatial and temporal variation, at each point, we made three spatial replicates each at a 1 m distance from a central area, as well as two temporal replicates within 40 s of the initial measure for each point measured, totaling six measurements per point (Fig. S2f). Because palms are scattered across the landscape and can generate microtopographic conditions, as well as present elevated respiration rates, the collars were installed at least 3 m away from any palm to minimize their influence on flux measurements. To standardize environmental conditions, sampling was performed between 10:00 a.m. and 2:00 p.m., when temperature and solar radiation peak. To mitigate the influence of vegetation disturbance, the rings were installed at least 48 hours before the first measurements and remained fixed until the end of field campaigns. Furthermore, all measurements were taken by the same person, who remained still near the rings for the sampling window.

Measures were taken using a LI-COR SmartChamber connected to an LI-7180 Trace Gas Analyzer. The chamber was opaque, with a diameter of 20 cm and a total volume of 4,244 cm³. The rings were installed to a depth of 2 cm, leaving approximately 10 cm above the soil surface. Because photosynthesis was not accounted for in the opaque chamber, all plant material inside the rings was removed 48 hours before the first sampling, and any resprouted vegetation in the following campaigns was removed prior to measurements. Sampling occurred across a 90 s interval, with a deadband of 20 s and a 60 s venting time between each measure. Fluxes were estimated by the SmartChamber software from the accumulation curve using the equation below:

$F_{C}= \frac{10VP_{0}(1 - \frac{W_{0}}{1000})}{RS(T_{0}+273.15)}*\frac{\delta C'}{\delta t}$

In which, the gas flux (F_c_) was based on the chamber volume (V), initial pressure (P_0_), molar fraction of water vapor (W_0_), gases universal constant (R), area measured (S), initial air temperature (T_0_) and variation of gases concentration in time ($\frac{\delta C'}{\delta t}$). We corrected the flows using water vapor to reduce the influence of air dilution within the chamber and the consequent underestimation of CO_2_ and CH_4_. The curves were processed using the SoilFluxPro software. During processing, the deadband length was adjusted to match the point at which fluxes stabilized and exhibited a constant slope. Measurements displaying evident bubbling effects were discarded.

**References**

Beer, F., Munhoz, C.B.R., Couwenberg, J., Horák-Terra, I., Fonseca, L.M.G., Bijos, N.R., Nunes da Cunha, C., Wantzen, K.M., 2024. “Peatlands in the Brazilian Cerrado: insights into knowledge, status and research needs.” Perspectives in Ecology and Conservation. https://doi.org/10.1016/j.pecon.2024.07.003

Beven, K.J., Kirkby, M.J., 1979. A physically based, variable contributing area model of basin hydrology / Un modèle à base physique de zone d’appel variable de l’hydrologie du bassin versant. Hydrological Sciences Bulletin 24, 43–69. https://doi.org/10.1080/02626667909491834

Burnham, K.P., Anderson, D.R., 2004. Multimodel Inference: Understanding AIC and BIC in Model Selection. Sociological Methods & Research 33, 261–304. https://doi.org/10.1177/0049124104268644

Dubois, C., Mueller, M., Pathe, C., Jagdhuber, T., Cremer, F., Thiel, C., Schmullius, C., 2020. Characterization of Land Cover Seasonality in Sentinel-1 Time Series Data, ISPRS Annals of Photogrammetry, Remote Sensing and Spatial Information Sciences. https://doi.org/10.5194/isprs-annals-V-3-2020-97-2020

Gislason, P.O., Benediktsson, J.A., Sveinsson, J.R., 2006. Random Forests for land cover classification. Pattern Recognition Letters, Pattern Recognition in Remote Sensing (PRRS 2004) 27, 294–300. https://doi.org/10.1016/j.patrec.2005.08.011

Google (2025) Google Earth [desktop software]. https://earth.google.com/

Hijmans, R.J., 2020. terra: Spatial Data Analysis. https://doi.org/10.32614/CRAN.package.terra

Kuhn M, Wickham H, 2020. Tidymodels: a collection of packages for modeling and machine learning using tidyverse principles. https://www.tidymodels.org.

Lewis, K., de V. Barros, F., Cure, M.B., Davies, C.A., Furtado, M.N., Hill, T.C., Hirota, M., Martins, D.L., Mazzochini, G.G., Mitchard, E.T.A., Munhoz, C.B.R., Oliveira, R.S., Sampaio, A.B., Saraiva, N.A., Schmidt, I.B., Rowland, L., 2022. Mapping native and non-native vegetation in the Brazilian Cerrado using freely available satellite products. Sci Rep 12, 1588. https://doi.org/10.1038/s41598-022-05332-6

Misra, G., Cawkwell, F., Wingler, A., 2020. Status of Phenological Research Using Sentinel-2 Data: A Review. Remote Sensing 12, 2760. https://doi.org/10.3390/rs12172760

Olofsson, P., Foody, G.M., Herold, M., Stehman, S.V., Woodcock, C.E., Wulder, M.A., 2014. Good practices for estimating area and assessing accuracy of land change. Remote Sensing of Environment 148, 42–57. https://doi.org/10.1016/j.rse.2014.02.015

Pebesma, E., 2016. sf: Simple Features for R. https://doi.org/10.32614/CRAN.package.sf

R Core Team, 2025. R: A Language and Environment for Statistical Computing. R Foundation for Statistical Computing, Vienna, Austria. https://www.R-project.org/.

Riihimäki, H., Kemppinen, J., Kopecký, M., Luoto, M., 2021. Topographic Wetness Index as a Proxy for Soil Moisture: The Importance of Flow-Routing Algorithm and Grid Resolution. Water Resources Research 57, e2021WR029871. https://doi.org/10.1029/2021WR029871

Roberts, D.R., Bahn, V., Ciuti, S., Boyce, M.S., Elith, J., Guillera-Arroita, G., Hauenstein, S., Lahoz-Monfort, J.J., Schröder, B., Thuiller, W., Warton, D.I., Wintle, B.A., Hartig, F., Dormann, C.F., 2017. Cross-validation strategies for data with temporal, spatial, hierarchical, or phylogenetic structure. Ecography 40, 913–929. <https://doi.org/10.1111/ecog.02881>

SICAR – Sistema Nacional de Cadastro Ambiental Rural, v3.47.1, 2023. https://www.car.gov.br/publico/municipios/downloads. Accessed on July 26, 2023.

Tucker, C.J., 1979. Red and photographic infrared linear combinations for monitoring vegetation. Remote Sensing of Environment 8, 127–150. https://doi.org/10.1016/0034-4257(79)90013-0

Valavi, R., Elith, J., Lahoz-Monfort, J.J., Guillera-Arroita, G., 2019. blockCV: An r package for generating spatially or environmentally separated folds for k-fold cross-validation of species distribution models. Methods in Ecology and Evolution 10, 225–232. https://doi.org/10.1111/2041-210X.13107

Wu, Q., Brown, A., 2022. whitebox: 'WhiteboxTools' R Frontend. R package version 2.2.0. https://CRAN.R-project.org/package=whitebox

Wu, J., Jones, K.B., Li, H., Loucks, O.L. (Eds.), 2006. Scaling and Uncertainty Analysis in Ecology. Springer Netherlands, Dordrecht. https://doi.org/10.1007/1-4020-4663-4
